# Unlocking regional bioeconomy transitions: Co-creation, stakeholder engagement, and systemic change in rural Europe

**DOI:** 10.12688/openreseurope.21341.1

**Published:** 2025-12-17

**Authors:** Margit Hofer, Martina Lindorfer, Samire Gurgurovci

**Affiliations:** 1 Zentrum fur Soziale Innovation, Vienna, Vienna, 1115, Austria

**Keywords:** Bioeconomy hubs, Co-creation, Stakeholder engagement, Conservative rural contexts, Innovation adoption, Trust-based governance

## Abstract

**Background:**

Transitioning to a sustainable bioeconomy in rural Europe is challenged by the difficulty of translating high-level strategies into locally relevant initiatives. Regional bioeconomy hubs aim to bridge this gap, but their success hinges on engaging diverse, often conservative, stakeholders. While co-creation is a promising approach, its practical application in these contexts is poorly understood. This study examines the establishment of nine hubs within the Horizon Europe project RuralBioUp to identify effective strategies for fostering stakeholder ownership and systemic change.

**Methods:**

This qualitative study analysed multi-layered data collected from nine regional bioeconomy hubs between October 2022 and June 2025. Sources included seven semi-structured interviews with hub facilitators, two mutual learning workshops, participatory feedback tools, and co-created documentation. Thematic analysis identified patterns related to effective actions, co-creation dynamics, and innovation adoption.

**Results:**

Effective engagement actions were practical and locally grounded, such as study visits, targeted training in local languages, and co-organized events. Generic online workshops and bureaucratic activities proved ineffective. Co-creation was enabled by institutional flexibility, trusted local facilitators, and established networks. Key barriers included stakeholder time scarcity, logistical challenges, cultural scepticism, and unclear value propositions. In conservative settings, innovation adoption was facilitated through peer-to-peer learning and framing new ideas as extensions of existing practices.

**Conclusions:**

The success of regional bioeconomy hubs is contingent on trust, perceived local relevance, and stakeholder ownership, not just technological novelty. Instead of one-size-fits-all models, patient, bottom-up strategies that co-adapt innovation to community values are essential for long-term change. Hubs can act as critical facilitators for place-based transitions by embedding co-creation within flexible governance structures. Policy and practice should prioritize context-sensitive, tangible engagement to foster inclusive and sustainable rural bioeconomy development.

## Plain Language Summary

Across Europe, many rural areas are trying to move toward a more sustainable economy that makes use of local natural resources instead of relying on fossil fuels. This shift is called the bioeconomy. To support this change, the RuralBioUp project (2022–2025) created nine regional bioeconomy hubs in different European countries. These hubs are spaces where farmers, businesses, researchers, community groups, and local authorities can come together to share ideas and create solutions that fit their region.

Our study looks at what worked well and what did not, when setting up and running these hubs. We found that people were most willing to participate when activities felt useful, practical, and close to their daily lives. For example, visiting real-life bioeconomy projects, holding training sessions in local languages, and organizing events with local partners made a big difference. In contrast, generic online workshops in English or activities with heavy paperwork often discouraged participation.

Trust and personal connections proved essential. Hubs led by local facilitators who knew the community built more credibility and involvement than those relying on outside experts. Success also depended on showing clear benefits, such as new funding opportunities for bioeconomy initiatives, recognition, or business contacts.

Introducing innovation in traditional rural areas takes time. People are more open to new ideas when they see them as a natural extension of what they already know, rather than as something completely new. Peer-to-peer learning for example, farmers learning from other farmers, was especially effective.

Overall, we conclude that bioeconomy hubs can become engines of sustainable rural development if they are locally grounded, trust-based, and adapted to community values and needs. Instead of one-size-fits-all approaches, patient, bottom-up strategies are key to long-lasting change.

## Introduction

As Europe transitions toward a sustainable, circular, and inclusive bioeconomy, rural regions play a pivotal role. They anchor bio-based solutions within local contexts. However, turning high-level strategies into actionable and regionally relevant initiatives remains a complex challenge.

The RuralBioUp project, funded under the Horizon Europe Framework, was designed to address this challenge. It supports the development and implementation of nine regional bioeconomy hubs across Europe.

Each hub operated in a unique socio-economic and environmental context. They selected two value chains and tailored their actions to local needs and opportunities. By fostering multi-stakeholder collaboration through co-creation, knowledge exchange, capacity building, and integrating the bioeconomy into regional development, the hubs collectively shaped a more resilient and locally adapted bioeconomy.

This work adds to the growing body of knowledge on how bioeconomy can be realized at the grassroots level. It also aligns with broader European objectives, such as the Green Deal, rural development, and the transition to climate-neutral societies.

Despite increasing policy attention to the bioeconomy as a driver of sustainable regional development, practical implementation of bioeconomy hubs remains challenging. Success depends heavily on the active involvement of diverse and often traditional - local stakeholders. These include public authorities, SMEs, farmers, regional developers, civil society, and knowledge institutions.

Co-creation has emerged as a promising approach for fostering inclusive engagement and developing context-specific solutions. Yet, little is known about how co-creation processes function in practice within regional bioeconomy settings. Questions remain about which conditions enable or hinder stakeholder participation, and which strategies effectively overcome common barriers.

This study provides empirical insights into these issues. It explores which actions are perceived as effective, how co-creation supports the establishment of regional bioeconomy hubs, and how implementation can succeed in traditional settings. The study presents key lessons from the implementation of these hubs between October 2022 and June 2025.

Using a multi-layered qualitative research approach - including semi-structured interviews, two mutual learning workshop outputs, co-created documentation, and participatory feedback tools - the study identifies patterns of success, persistent challenges, and transferable practices in rural bioeconomy development.

Research Questions

This study addresses three research questions:

- RQ1: Which actions are perceived by local facilitators as effective or ineffective in establishing regional bioeconomy hubs?
- RQ2: What systemic and contextual conditions facilitate or hinder the practical implementation of co-creation in developing regional bioeconomy hubs?
- RQ3: What strategies have proven effective in initiating mindset shifts and enabling the adoption of bioeconomy and innovation in traditionally structured or conservative rural environments?

Through this work, we contribute to the growing understanding of how bioeconomy can be realized at the grassroots level. We offer a nuanced view of how collaborative innovation processes can be operationalized in regional bioeconomy contexts.

Our findings build on evidence from earlier initiatives such as BIOVoices ^1^ and BioBridges ^2^. While those projects mainly focused on knowledge sharing and policy dialogue, RuralBioUp goes further. It demonstrates concrete co-creation strategies that foster long-term stakeholder ownership, even in conservative rural settings.

The novelty of this study lies in combining empirical evidence from nine regional hubs with a focus on co-adaptive approaches. In doing so, it fills a gap in the bioeconomy hub literature.

The findings aim to guide both future research and practical efforts. They offer strategies for designing effective stakeholder engagement and implementation frameworks for sustainable bioeconomy initiatives. These efforts align with broader European goals such as the Green Deal, rural revitalization, and the transition to climate-neutral societies.

This paper therefore outlines the theoretical framework and the RuralBioUp hub concept. It then describes the research methodology, presents the key findings from the multi-layer qualitative analysis, and discusses their implications for regional bioeconomy development. Finally, it concludes with broader lessons and future directions for policy and practice.

## Conceptual framework

The transition toward a sustainable and circular bioeconomy in Europe requires more than technological innovation. It also demands profound social, institutional, and cultural change, especially in rural regions. High-level strategies and funding mechanisms exist to promote bio-based solutions. However, their effective implementation on the ground depends heavily on local engagement, institutional readiness, and socio-cultural context. The RuralBioUp project, through the creation of nine regional bioeconomy hubs, provides a valuable lens into real-world processes. It shows how bioeconomy innovation can be initiated and sustained in diverse rural settings.

This chapter establishes the theoretical foundation for analyzing the three central research questions of the study. First, it examines how local actors perceive specific actions as effective or ineffective in establishing regional bioeconomy hubs (RQ1). Second, it investigates the conditions - both facilitators and barriers - that shape the translation of co-creation theory into practice (RQ2). Third, it explores the strategies that have proven effective for introducing bioeconomy and innovation in traditionally structured or conservative environments. In such contexts, resistance to change and established mindsets can hinder the adoption of innovation (RQ3). To address these questions, the conceptual framework draws on interdisciplinary literature. It integrates insights from rural development, innovation studies, transition theory, and co-creation approaches. By combining these perspectives, the chapter highlights the interplay between structural conditions, cultural norms, and institutional environments. This interplay determines how bioeconomy implementation unfolds in practice. The goal is to identify where empirical findings from the RuralBioUp project can enrich scholarly understanding. In particular, the study contributes to knowledge on participatory innovation and system change in rural contexts.

### Bioeconomy in local/regional contexts

Consequently, bioeconomy in a rural setting can be understood as a place-based innovation strategy that builds on the unique assets, knowledge, and capacities of specific regions to drive sustainable economic development ^3, 4^. Rather than applying uniform solutions, approaches require to be adapted or tailored to each region’s ecological, economic, and socio-cultural context ^5^.

Thus, innovation connects to the local context by acknowledging the availability of natural resources in the region. It also considers the potential of cultural practices, institutional arrangements, and existing stakeholder networks. Recent empirical work underscore *“… the importance of participative approaches as key to the development of bioeconomy strategies, for which a key issue is the active engagement of a great diversity of stakeholders and the establishment of an inter-and cross-sectoral, as well as inter- and transdisciplinary cooperation in the region”* ^6, 7^.

In addition, spatially sensitive approaches are also essential. They ensure that innovation strategies respond to the territorial and ecological characteristics of rural regions ^6, 8^. This makes us understand that successful bioeconomy transitions often rely on several dimensions and perspectives. They also depend on interdependencies of regional embeddedness which foster social sustainability and resilience ^5, 6, 9, 10^. Thus, bioeconomy has developed during the past years more than just following traditional pathways of production with sustainable materials. It includes also the change of the society towards a sustainable development along the entire value chain ^11– 13^. It also highlights the need to provide guidance that will allow stakeholders like farmers, local developers, researchers, policymakers, government, and business to develop the way forward towards sustainable innovation.

Thus, a holistic and multidisciplinary vision that is able to cope with the complexity of circular bioeconomy along the entire value chain including its different stakeholders is highly complex. This is due to the interplay of diverse sectors, fragmented governance structures, conflicting policy objectives, regional differences, and the need to integrate environmental, economic, and social sustainability considerations ^14– 16^.

To sum up, together, these findings provide robust theoretical and empirical grounding for the argument that rural bioeconomy hubs must operate as place-based, context-aware initiatives. Rooted in co-creation and participatory governance, they can realize locally relevant, resilient, and sustainable outcomes.

### Bioeconomy hubs as incubators for systemic change and local adaption

Fostering this systemic shift, the practical implementation has been enforced by bioeconomy hubs.

Bioeconomy hubs can serve as facilitators of systemic change and local adaptation by catalysing multi-stakeholder collaboration. They align innovation to regional contexts and embed circular bioeconomy solutions within existing regional socio-ecological potentials and opportunities ^6, 17, 18^.

These hubs can act as innovation-incubators orchestrating regional stakeholders’ needs and requirements, such as primary producers, regional developers, public authorities, SMEs, research institutions, and civil society. Often the value chains use the (local) knowledge of these stakeholders to adapt to local biomass availability, infrastructure, and policy conditions.

In this regards, empirical studies demonstrate that hubs build dynamic capabilities ^10^. These include the abilities to sense, seize, and adapt towards a knowledge-based bioeconomy.

Other case studies emphasize how district-level bioeconomy strategies (created through stakeholder workshops and quadruple-helix governance) are operationalized locally. This ensures context-sensitive implementation ^10, 18^.

Moreover, the concept of systemic innovation, characterized by cross-sector stakeholder integration and collaboration, emerges as a crucial mechanism for operationalizing the bioeconomy across value chains. It enables coordinated transitions that align economic, environmental, and social objectives ^19– 22^.

Circular bioeconomy in specific can also act as a driver of system integration. It unites agriculture, forestry, aquaculture, and innovation into cohesive regional systems ^15^.

From a governance perspective, hubs can be understood as intermediaries that create connections, reduce coordination failures, and accelerate transitions. This perspective links bioeconomy hubs to broader transition studies and intermediary typologies [Kivimaa19]. Likewise, the notion of hubs as living labs resonates with the literature on participatory and experimental innovation environments ^23^, which emphasizes their role as structured spaces for testing, co-creation, and diffusion.

Collectively, this literature confirms that bioeconomy hubs are more than demonstration centres. They are local connectors that support and speed up local transition and reinforce regional resilience, adaptability, and sustainable innovation.

In line with the given literature, the nine RuralBioUp hubs defined the hubs as *“…collaborative space and facilitation tool that brings together diverse stakeholders - such as entrepreneurs, researchers, innovators, policy makers, civil society, media, and other regional actors - across the entire bioeconomy value chain. Its purpose is to foster dialogue, knowledge exchange, and networking in order to identify synergies, share best practices, and co-create solutions to regional and cross-regional challenges and opportunities in the bioeconomy. Through training, consulting, and strategic collaboration, bioeconomy hubs aim to build capacity, influence policy, and support the effective and sustainable use of renewable biological resources. Ultimately, they contribute to the development of functioning bioeconomy systems that align with broader goals such as regional development, climate neutrality, and the European Green Deal. The implementation differs highly from the regional identified opportunities, chances and cultural working modes.”* (Co-created bioeconomy hub definition, RuralBioUp consortium, Brussels May 2025)

This definition of RuralBioUp hubs positions them as incubators for systemic change and local adaptation. By intentionally convening a wide spectrum of stakeholders across the bioeconomy value chain - including not only entrepreneurs and researchers but also civil society and media - the hub becomes a structured space for cross-sectoral experimentation and learning. This fosters the emergence of new governance modes, business models, and innovation pathways. These can challenge and gradually replace traditional, linear economic systems. Moreover, the emphasis on dialogue, co-creation, and knowledge exchange indicates a commitment to adaptive, participatory processes. These are essential for managing the complexity of systemic transitions. The inclusion of regional variation - acknowledging that implementation “differs highly” based on local opportunities and cultural modes - further reinforces the hub’s function as a locally embedded, flexible mechanism. It is capable of responding to unique socio-economic and environmental contexts. Training, consulting, and strategic collaboration serve as mechanisms not only for capacity building but also for embedding new practices and norms into regional systems. In this way, they contribute to both structural transformation and context-sensitive innovation. Ultimately, the hubs act as dynamic interfaces and serve as engines for place-based systemic change.

### Multi-stakeholder integration via co-creation in bioeconomy development

The request for holistic strategies calls for inclusive, participatory approaches. In this context, participatory methods like co-creation and multi-stakeholders aligns well with the need of aligning bioeconomy development with societal adaptation, local requirements, and sustainable implementation.

The term ‘multi-stakeholder’ refers to the intentional inclusion and engagement of diverse actor groups (such as public authorities, businesses, academia, civil society, and citizens) in collaborative processes of problem framing, decision-making, implementation, and evaluation. Multi-stakeholder approaches aim to integrate plural perspectives, build legitimacy, and enhance the relevance and acceptance of innovation and governance outcomes ^24, 25^. In the context of bioeconomy development, multi-stakeholder processes are particularly valuable for aligning innovation strategies with local needs. They are also crucial for managing complexity across sectors and fostering systems thinking ^26^. This approach requires principles of participatory governance and responsible research and innovation (RRI). RRI promotes anticipatory, reflexive, and inclusive approaches to innovation that align with societal values and address grand societal challenges ^27– 29^.

Building on multi-stakeholder engagement, co-creation provides a structured approach for these diverse actors to actively collaborate and jointly shape solutions through shared learning and iterative knowledge integration. Co-creation is a collaborative process in which these diverse actors - such as farmers, local developers, researchers, entrepreneurs, policymakers, and civil society - jointly define problems and develop solutions through mutual learning and knowledge integration. It involves stakeholders throughout the innovation cycle and emphasizes inclusiveness, reflexivity, and context sensitivity ^30, 31^. In sustainability and bioeconomy contexts, co-creation fosters shared ownership and system-level learning. This makes it essential for effective and locally embedded innovation ^32, 33^. Rooted in innovation systems theory and transition theory e.g. 22, 34, co-creation emphasizes the systemic nature of innovation. Knowledge is produced not only by experts but also through experiential, contextual, and tacit knowledge held by practitioners. This inclusive engagement of stakeholders spans the entire innovation lifecycle, from agenda-setting and design to implementation and finally evaluation.

The inclusion of multi-stakeholders plays a vital role in the co-creation approach. Only the integration of the broad spectrum of all societal actors, including those traditionally excluded from innovation processes, will ensure that outputs are relevant to all stakeholders. This approach is increasingly institutionalized in EU bioeconomy policy frameworks, such as through the concepts of Responsible Research and Innovation (RRI) and Mobilisation and Mutual Learning (MML). EU bioeconomy policy itself strongly emphasizes multi-actor engagement as a foundation for sustainable development, framing participation as a selling point of the policy agenda ^35^.

While RRI promotes anticipatory, inclusive, and reflexive research and innovation processes that align with public values and address grand societal challenges ^28^, MML emphasizes horizontal learning, trust-building, and co-production of knowledge among diverse stakeholders. These are crucial for overcoming scepticism and fostering legitimacy in traditional or conservative contexts. Social learning is therefore a crucial component, since it enables actors to collectively reflect, adapt, and embed new practices in governance systems ^24^. Other bioeconomy hubs like BIOVoices ^1^ or BioBridges ^2^ that chose this format to co-develop knowledge, policy recommendations, and innovation pathways exemplify this approach. They catalyse stakeholder dialogue, align innovation strategies with societal values, and translate shared challenges into collaboratively designed action plans. ICLEI’s ^36^ implementation of MML further demonstrates how such processes build trust, legitimacy, and shared policy ownership across regions ^36^

Despite this promising approach, co-creation faces significant requirements. Along with this, it also faces barriers like trust, motivation, or imbalance of stakeholder issues, to name some of the most important ones. Trust is a foundational condition for meaningful engagement. A lack of trust will hinder true participatory processes ^37– 39^. Also, motivation is a central element of participatory approaches. Motivation is widely recognized as a central driver in participatory processes. Stakeholders will only dedicate their limited time and energy when they perceive tangible relevance, benefits, and a genuine sense of ownership in the initiative. Equally important in participatory governance is the balanced distribution of knowledge, interests, and representation across stakeholders. This is necessary to prevent power imbalances. Design elements aimed at inclusion are essential to avoid power imbalances that distort deliberation and marginalize vulnerable stakeholders. They highlight that unchecked disparities in instrumental, structural, or discursive power can undermine collaboration and social learning in governance processes. In rural bioeconomy settings, power imbalances are common. Local actors often follow established hierarchies or lack the resources to engage equally with researchers or government officials. More broadly, sustainability transition scholars emphasize that shifts in power relations are central to enabling systemic change ^40^.

To overcome these challenges, bioeconomy hubs and regional facilitators must adopt deliberate strategies to support mindset change, build social capital, and enable peer-to-peer activities. Structured methods such as deliberative workshops, living labs, and stakeholder roundtables can create spaces where actors safely express concerns, negotiate priorities, and build shared visions. Systemic innovation driven by multi-actor collaboration and stakeholder alignment is essential to operationalizing the bioeconomy across value chains. It facilitates coherent transitions that balance economic, environmental, and social objectives ^41, 42^.

Ultimately, co-creation in the bioeconomy is not merely about consultation. It is a transformative practice that reshapes the very foundations of how innovation is understood, governed, and implemented. When designed thoughtfully, multi-actor approaches can foster deeper legitimacy, adaptability, and impact in regional bioeconomy transitions, particularly in traditional and risk-averse settings.

### Co-creation in relation to perceived effectiveness of actions

The implementation of regional bioeconomy hubs cannot be evaluated solely by technical design quality or alignment with high-level policy goals. Rather, the perceived effectiveness of actions - how they are interpreted, experienced, and valued by local stakeholders - is essential for their uptake, and sustainable implementation. Co-creation, by facilitating inclusive, place-based development, directly enhances this perception of effectiveness and strengthens ownership of stakeholders.

Recent literature stresses that co-creation processes involving multiple stakeholder groups improve both, the relevance and credibility of regional innovation strategies ^26^. Through early and ongoing involvement in agenda setting, planning, and implementation, local actors can express their context-specific needs, expectations, and constraints. This embeddedness fosters interventions that stakeholders perceive as more meaningful, timely, and legitimate, even when conventional performance metrics such as number of events or pilot projects might suggest only modest progress ^30^. Importantly, perceived effectiveness is not merely a subjective opinion. It influences real behaviours, including the willingness of actors to collaborate, invest effort, and sustain initiatives beyond the funding period ^40^. If actors view hub activities as aligned with their local reality and values, they are more likely to mobilize their networks and resources in supporting them.

Local ownership emerges as a fundamental mechanism through which co-creation enhances both perceived and actual effectiveness. When local actors shape not just goals but also the design and delivery of hub activities, they gain a sense of agency and control over outcomes. This aligns with Arnstein’s classic notion of participation as empowerment through citizen control ^43^ and with more recent insights on collaborative governance emphasizing shared authority and legitimacy ^44, 45^. A participatory approach fosters empowerment and inclusivity. It aligns with findings that co-creation methods redistribute power, amplify participant voices, and ensure accessibility for diverse populations ^46, 47^. This fosters intrinsic motivation, strengthens emotional and cognitive investment, and increases the likelihood that new practices will be maintained over time. Ownership also enhances social capital. This is particularly important in rural areas where trust, reciprocity, and informal networks play a significant role in mobilizing action. In such settings, accounting for stakeholder engagement while acknowledging and appreciating their fundamentally different judgements, value frames, and viewpoints is vital ^46^. Co-created initiatives are more likely to reflect local narratives and leverage existing capacities. This contributes to higher uptake and reduced friction.

Beyond initial engagement, co-creation processes that include local actors in monitoring, evaluation, and adjustment of hub activities further strengthen trust and transparency. This dynamic feedback-driven governance supports adaptive implementation. It allows for the refinement of goals and methods in response to evolving conditions or unforeseen barriers ^26^. For example, in the RuralBioUp project, regional co-creation hubs surfaced early barriers (via SWOT analysis) such as lack of bioeconomy knowledge, limited innovation mindset, or fragmented stakeholder interest. These insights enabled timely adaptations and the development of solutions that were locally relevant and thus more readily accepted.

In rural development, the context-specific nature of challenges - such as remoteness, limited infrastructure, or path-dependent institutional practices - often make standardized models of implementation ineffective.

Co-creation enables embedding in the local context. Hub strategies are continuously adapted to local realities through direct interaction with stakeholders. This adaptability is essential for bioeconomy hubs, which must navigate diverse environmental, economic, and social systems. Co-creation builds in the flexibility required to respond to local constraints and opportunities. The process itself becomes a contributor to perceived effectiveness. Stakeholders value not just the outcomes, but the degree to which the process accommodates their input, adapts to change, and respects local knowledge and capacity.

In sum, the perceived effectiveness of regional bioeconomy hub implementation is not simply an outcome. It is a product of how implementation via participation is conducted. Co-creation improves perceived effectiveness by enhancing the effectiveness of actions. It supports the building of local ownership and motivation, facilitates adaptation to local needs, and fosters trust, learning, and sustained commitment. Embedding bioeconomy hubs in co-created, participatory processes is therefore not only a normative ideal but also a strategy for increasing their effectiveness, resilience, and long-term viability ^44, 48^.

### Innovation as a systemic shift in traditional and conservative settings

Innovation in rural and traditional settings is not merely a matter of introducing new technologies or practices. It demands a systemic shift that fosters transformation at institutional, cognitive, and social levels ^16^. Rural areas often follow established paths shaped by historical routines and entrenched institutions, making it difficult to adopt new approaches ^10^. Innovation is less about disruption and more about navigating and reshaping these complex systems through agency, adaptive governance, and networked strategies. Such processes resonate with the multi-level perspective on socio-technical transitions, which highlights how innovations emerge in niches and gradually reshape existing regimes under broader landscape pressures ^22, 49^. Innovation is less about disruption and more about navigating and reshaping these complex systems through agency, adaptive governance, and networked strategies ^50^.

Empirical research ^51^ underscores that rural innovation often builds on strong local networks and social capital. It relies on incremental path development and interactive governance rather than radical technological change. Effective transformation in such contexts depends on bridging roles, such as network enablers and knowledge brokers, to cultivate adaptive governance and transformative social innovation. Ultimately, rural innovation must embrace systemic approaches that respect historical path-dependencies, leverage community agencies, and operate within socio-technical regimes to realize sustainable and locally embedded outcomes. Grassroots innovations are particularly relevant here, as they highlight how communities can generate bottom-up solutions aligned with local values and practices ^52^.

From an economically driven approach, a systematic review of high-quality studies ^53^ identified four key dimensions that consistently drive the adoption of green innovation. They refer to government policies and regulations, technological capabilities (availability, affordability, and suitability), organisational culture and HR practices, and market dynamics including consumer demand. Additional influences include economic incentives, environmental concerns, strategic orientation, firm characteristics, and perceived environmental risks. Overall, green innovation adoption is shaped by a dynamic interplay of institutional, technological, organisational, and market factors.

On a socio-economic level, the Diffusion of Innovations theory ^54^ offers a valuable lens to understand how innovation adoption unfolds within society. Rogers ^54^ defines diffusion as the process by which an innovation is communicated over time through specific channels within a social system. An innovation refers to an idea, practice, or object that is perceived as new by an individual or adoption unit. The diffusion process involves both mass media and interpersonal communication channels. Rogers identifies key adopter categories (from innovators and early adopters to experts) and emphasizes how factors such as perceived relative advantage, compatibility with existing values, and complexity influence the rate and success of adoption.

In traditional rural contexts, many stakeholders fall into the more cautious adopter categories. This reflects a general risk aversion, limited exposure ^55^ to external innovation networks, and reliance on established practices. These conditions can create resistance to change, particularly when innovations are perceived as externally imposed or disconnected from local priorities and experiences.

Addressing these challenges requires targeted strategies to foster mindset change, trust-building, and peer-to-peer learning. Mindset change is often a slow process. It is shaped by repeated exposure to innovation narratives, visible local success stories, and the gradual erosion of scepticism. Trust-building is equally essential, particularly in contexts where top-down initiatives have historically failed or created dependency. In this regard, bioeconomy hubs play a critical intermediary role. They act as facilitators that create safe spaces for experimentation, dialogue, and participatory co-creation, thereby aligning innovation processes with local values and lived realities.

Additionally, peer-to-peer learning has emerged as one of the most effective mechanisms for overcoming cognitive and cultural barriers to innovation adoption. When farmers, entrepreneurs, or community leaders observe their peers successfully implementing bio-based practices - such as circular farming models, cooperative biomass valorisation, or new forms of regional marketing - they are more likely to reconsider their own positions. Such horizontal learning builds both legitimacy and trust, especially when reinforced by existing social networks and local (highly influential) stakeholders who can act as innovation brokers within their communities. Empirical studies confirm that regions with strong social capital and embedded peer networks exhibit higher openness to rural innovation ^55, 56^.

Thus, fostering innovation in traditional settings is not only a matter of technical or economic introduction. It is also a matter of social embedding, requiring time, dialogue, and a deep understanding of local dynamics.

### Synthesis: framing the research questions

This study adopts an interdisciplinary framework combining innovation theory, co-creation, rural sociology, and bioeconomy implementation. It examines how regional bioeconomy hubs can facilitate systemic transformation in rural areas. Recognizing that the shift to a circular bioeconomy entails not merely technological but also institutional and socio-cultural change, the research positions bioeconomy hubs as place-based innovation infrastructures. These hubs mobilize local resources, foster cross-sectoral collaboration, and respond to region-specific needs. Co-creation is treated as both a theoretical and practical approach to inclusive innovation. It is grounded in transition studies, innovation systems, and participatory governance. Co-creation plays a key role in enhancing perceived effectiveness, fostering local ownership, and increasing the legitimacy and sustainability of interventions. The study addresses a critical gap between policy aspirations and on-the-ground practices in rural bioeconomy development, particularly in conservative or traditionally structured regions. It investigates three core research questions:

1. RQ1 explores stakeholder perceptions of what actions are effective or ineffective in establishing bioeconomy hubs, linking perceived effectiveness to engagement and implementation outcomes.
2. RQ2 examines enabling and constraining conditions for translating co-creation theory into practice, identifying real-world challenges such as power imbalances, institutional inertia, and trust deficits.
3. RQ3 investigates strategies for introducing innovation in traditional rural settings, emphasizing co-adaptation over disruption and drawing on trusted networks, peer learning, and visible local benefits.

Through these questions, the study contributes to a deeper understanding of rural innovation processes, offering empirical insights for improving stakeholder engagement, hub implementation, and socially inclusive bioeconomy transitions. The findings are intended to inform both academic discourse and policy practice.

### The RuralBioUp hubs framework

The understanding of hubs can differ dramatically in terms of goals, functions, aims, and objectives.

As for the RuralBioUp bioeconomy hubs, the partners of the project collaboratively defined the hubs as a collaborative space and facilitation tool. These hubs bring together diverse stakeholders - such as entrepreneurs, researchers, innovators, policymakers, civil society, media, and other regional actors - across the entire bioeconomy value chain. Their purpose is to foster dialogue, knowledge exchange, and networking. They aim to identify synergies, share best practices, and co-create solutions to regional and cross-regional challenges and opportunities in the bioeconomy. Each RuralBioUp hub is facilitated by a hub facilitator who coordinates and implements hub activities to drive innovation and collaboration among stakeholders. They guide the process by fostering communication, managing resources, and ensuring effective participation throughout the innovation cycle. Through training, consulting, and strategic collaboration, bioeconomy hubs aim to build capacity, influence policy, and support the effective and sustainable use of renewable biological resources. Ultimately, they contribute to the development of functioning bioeconomy systems that align with broader goals such as regional development, climate neutrality, and the European Green Deal ^57^. The implementation can differ highly depending on the region’s identified opportunities, chances, and cultural working modes.

## Methodology

The definition of the hubs frames the research field as a collaborative space and facilitation tool that brings together diverse stakeholders across the entire bioeconomy value chain. This co-created definition outlines the core objectives and functions of the hubs while also reflecting the approach for implementation and types of actions undertaken.

To ensure participant confidentiality, all data collected from the hubs were anonymized. However, the population size of the respective countries shapes the diversity, scale, and complexity of stakeholder environments. Thus, it may influence how findings are interpreted. Country size is particularly relevant to stakeholder dynamics, as it affects the institutional landscape and network configurations in which hubs operate. In larger countries, stakeholder environments are often more fragmented and bureaucratically layered, which may pose coordination challenges. In contrast, smaller countries tend to have denser interpersonal networks and closer institutional proximity, which can facilitate faster trust-building and stakeholder alignment. To provide contextual insight, Table 1 categorizes the participating hubs according to the population size of their respective countries.

**Table 1.** Hub codes and size of respective country.

Code for Interviewed Hub	Country Size>
H1 (covering two hubs)	Each large population, >50 million
H2	Medium Population (10–20 million)
H3	Medium Population (10–20 million)
H4	Small Population (1–10 million)
H5	Small Population (1–10 million)
H6	Large population, >50 million
H7 & H8	Large population, >50 million

To extract findings from the implementation of the RuralBioUp hubs, we applied a comprehensive, multi-layered analysis covering the entire project duration from its launch in October 2022 through to June 2025.

A qualitative approach was chosen to explore the complex, context-dependent experiences and perceptions of hub facilitators involved in the RuralBioUp project. Therefore 7 semi-structured interviews (two of them as group interviews) with hub facilitators were done in spring 2024, covering all 9 hubs from regions in Italy (Lombardy, Marche Bioeconomy Hub, Puglia Bioeconomy Hub), France (Auvergne-Rhône-Alpes Bioeconomy Hub, Pays de la Loire & Bretagne Bioeconomy Hub), Romania (Centru Region Bioeconomy Hub), Czech Republic (Charles Spa Bioeconomy Hub), Ireland (Tipperanry Bioeconomy Hub) and Latvia (Latvia Bioeconomy Hub).

Given the diversity of regional settings, the evolving nature of bioeconomy initiatives, and the importance of capturing both successes and challenges in implementation, qualitative methods were best suited to uncover in-depth insights that would not be accessible through purely quantitative approaches. This approach allowed us to understand not only what happened in each hub, but how and why certain dynamics unfolded. The decision on qualitative methods resulted in providing a rich, nuanced foundation for deriving transferable lessons and policy recommendations. Although the study focused on seven interviews covering nine hubs, the diversity of regional, socio-economic, and cultural contexts represented across multiple EU member states provided sufficient variation to generate transferable lessons for rural bioeconomy development.

**Figure 1.**
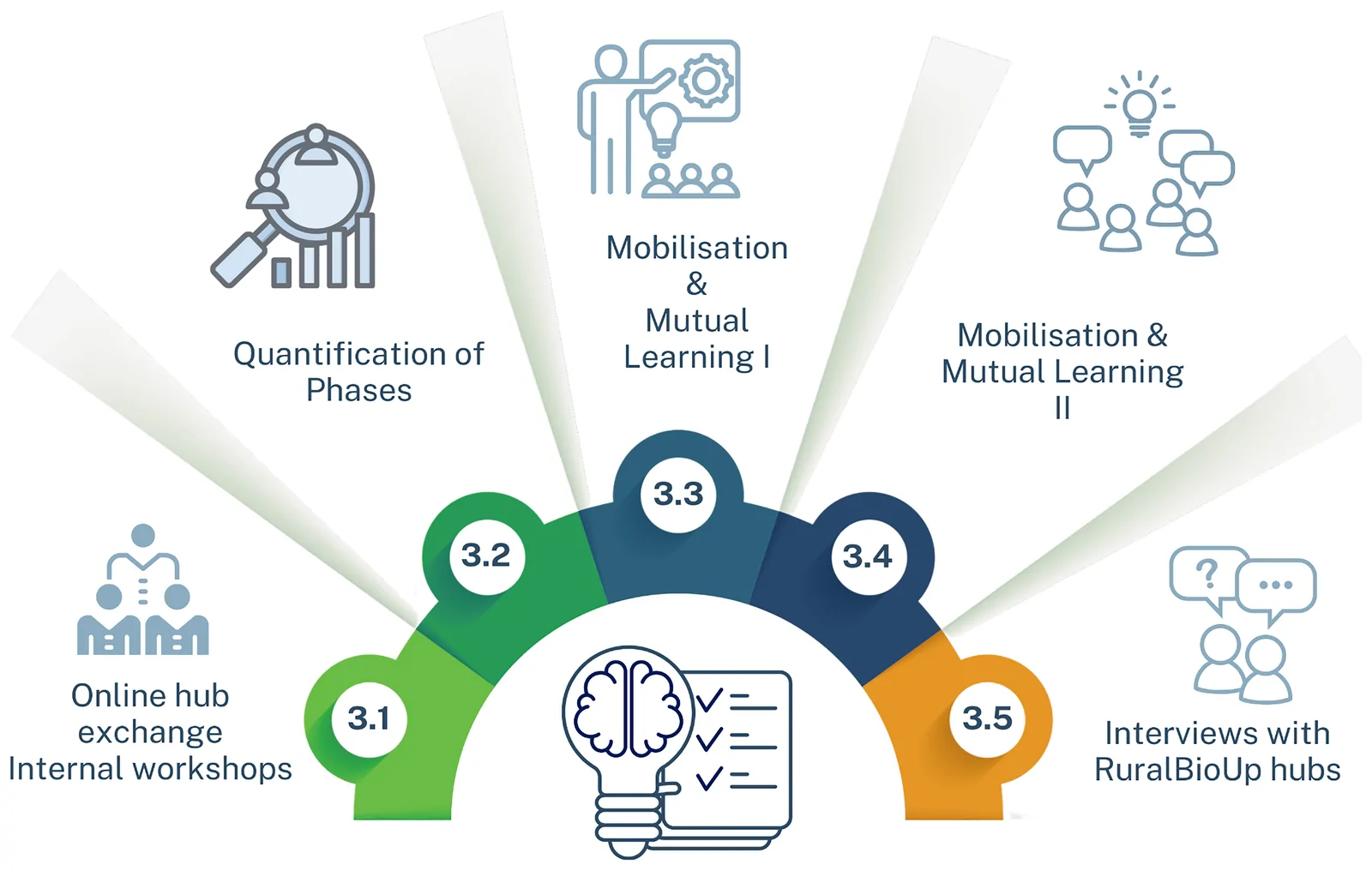
Overview sources for qualitative analysis. The analysis drew on multiple, complementary data streams to capture diverse stakeholder perspectives and operational dynamics. By combining structured workshops, phased assessments, multi-actor meetings, interviews, and on-site exchanges, the study ensured triangulation and contextual depth in understanding hub activities.

The qualitative data collection provided a large number of sources ( Figure 1) guiding the entire process of planning, implementing and evaluating the bioeconomy hubs. The diversity and volume of inputs - ranging from online hub exchange meetings, two mutual learning (MML) workshops, one interactive ranking via Mentimeter to seven semi-structured interviews with the hub facilitators of the nine regional project hubs, allowed for a well-rounded and in-depth understanding of the hub’s dynamics.

This multi-method approach enabled the capture of both structured feedback (via interviews, exchange meetings, and workshops) and more spontaneous insights (via ranking via Mentimeter sessions). The combination of these data sources provided a well-rounded view of the project's achievements, challenges, and areas for improvement.

The collected qualitative data sets of the interviews were analysed using MAXQDA, a qualitative data analysis software widely used in social sciences. MAXQDA enabled us to systematically code and categorise qualitative inputs to identify recurring themes, patterns, and insights. In qualitative research, coding refers to the process of organising and labelling segments of textual data based on emerging themes or concepts. These codes help researchers interpret the data systematically and uncover underlying structures or meanings. The software's advanced features allowed for the integration of multiple coding lists. For the analysis, we started with 9 overall deductive codes (pre-defined based on theory or research questions) derived from the online hub exchange meetings. These overall codes were further classified in total of 36 inductive codes (developed for themes that emerged during the coding process) according to relevant dimensions, offering a nuanced understanding of aspects that influenced the implementation, governance, socio-economic functionalities, issues and facilitating aspects of the RuralBioUp hubs. This process ensured consistency of defined key topics as well as the flexibility for new, emerging topic areas within a structured analytical process.

Our analysis of the data sets resulted in a total of 240 coded segments, capturing a wide spectrum of insights gathered throughout the study. The research results derived from the qualitative analysis were further refined, detailed, and verified through two MML workshops. The MML workshops enabled cross-regional comparison, clarification of ambiguous findings, and identification of additional contextual factors that had not surfaced during individual interviews. They also supported the triangulation of results and reinforced the credibility and relevance of the emerging categories by allowing participants to confirm, nuance, or challenge early interpretations.

## Results and findings

The empirical findings in response to the three research questions, focusing on: (RQ1) hub facilitators perceptions of effective and ineffective actions in establishing bioeconomy hubs; (RQ2) the systemic conditions that facilitate or hinder the practical implementation of co-creation; and (RQ3) strategies for fostering innovation and initiating mindset shifts in traditionally structured rural environments revealed each several aspects that are outlined in the following section:

### Hub facilitators perceptions of effective and ineffective actions for establishing regional bioeconomy hubs (RQ1)

Based on qualitative feedback the overall finding clearly stresses the importance of engagement and sustained participation of local actors and emphasizes that it requires more than well-intended policy design or funding. Understanding which actions are perceived as effective or ineffective is essential for translating strategic intentions into operational success. Thus, effective strategies centred on practical, tangible benefits and direct, personal engagement, such as study visits and targeted, co-organized events. In contrast, actions perceived as too generic, administratively burden or lacking clear, immediate value, were less effective at securing stakeholder participation and commitment.

***The importance of perceived value of participation by participants***

Accordingly, to the hub facilitators, stakeholder engagement is not only influenced by the theoretical relevance or strategic ambition of an action, but by its perceived practical value. Local actors (entrepreneurs, farmers, SME representatives, or municipal officers) evaluate initiatives based on immediate relevance, tangible benefits, and the degree to which they feel directly addressed. This lived experience perspective helps explain why some interventions succeed in generating commitment in some hubs while others did not. Effective RuralBioUp actions were typically those that felt hands-on, directly useful, and personally meaningful, while ineffective ones were often perceived by the hub facilitators as abstract, burdensome, or misaligned with local needs and capacities.

***Effective actions: what worked according to hub facilitators***

**Practical and Tangible Activities**

A consistent theme in hub facilitators’ feedback was the importance of concrete, experiential learning formats. These were perceived as much more compelling than abstract presentations or theoretical sessions:

The study visits (visits of hub members to another exchanging bioeconomy hub or facility) emerged as a particularly powerful engagement tool. Study visits participants described them as valuable opportunities stating that “ *This is one of the best ways to include and share information and to be practical. Because we included in our approach methodologies based on learning by doing, learning by seeing, and learning by touching*” (H7). This suggests that allowing hubs to interact directly with hub members on real-life examples of bioeconomy practices is a very effective action. These visits helped demystify bioeconomy concepts and inspired replication by showing local actors what is realistically achievable. Participants emphasized that study visits were impactful because they provide information about the facility on the ground so that people could get a better understanding of what it was previously, what it is currently and what we hope to do in the future (H4).

Business matchmaking events that connected biomass producers with potential users of their by-products were highly appreciated too. These formats were seen as immediately useful, providing tangible economic opportunities and fostering new collaborations for business and innovation development.

Events that fostered visibility and recognition also played a significant motivational role. According to the facilitators, the hubs stakeholders responded positively to being featured in promotional materials or invited to speak at events. Such exposure was viewed not only as a form of appreciation but also as a way to build professional reputation and position oneself as a pioneer in the regional bioeconomy field.

**Targeted and Collaborative Events**

The format and context in which events were organized had a strong influence on participation and overall impact:

Co-organizing events with other ongoing European or local initiatives enhanced legitimacy and visibility: “ *Co-organizing worked well. It attracted more participants and allowed for broader engagement.”* (H1) The hub facilitators reported that such collaborative formats felt less isolated, top-down interventions and more like integrated community efforts. Participation was significantly higher when events were embedded in existing networks and activities, rather than being project exclusive.

On-site training sessions on specific, pre-identified topics (e.g. information on legal treatment of by-products) proved to be particularly successful. Effectiveness was enhanced when these sessions were delivered in the local language, allowing participants to fully engage with the content and ask questions in a familiar context. The specificity and contextual relevance of the topics were key factors in maintaining attention and promoting uptake.

Direct, personal outreach methods also proved highly effective, particularly in more conservative or traditionally structured areas. *“A ‘door-to-door’ engagement approach was effective in the hub’s country, directly visiting businesses instead of relying on emails that might get lost in spam.”* (H7) This outreach method enabled the building of personal trust, reduced perceived distance between project teams and community members, and helped initiate conversations that might not have occurred through formal invitations alone.

**Strategic Networking and Support Mechanisms**

Beyond individual activities, effective engagement also relied on leveraging existing structures and addressing core stakeholder needs:

Utilizing established networks, such as farmer associations, regional development agencies, or cooperative groups, was critical for building legitimacy and ensuring outreach success. These intermediaries helped disseminate information more credibly and connected project teams with harder-to-reach stakeholders. They acted as trusted bridges between innovation agendas and local communities: *“Success hinges on the collaboration of local authorities, academia, industry, and civil society, driven by innovation”* (H2)

Providing access to information on funding opportunities was a powerful motivator accordingly to hub facilitators. Across regions, stakeholders expressed a consistent interest in learning how their involvement in hub activities could lead to financing opportunities for their own projects or business ideas. The perception that participation could unlock financial support was a strong driver of engagement.

***Ineffective actions: what didn’t work - and why***

While several strategies succeeded in building trust and engagement, the hub facilitators also identified a number of actions that were perceived as ineffective or counterproductive. These insights are equally valuable, as they highlight common pitfalls and reveal how certain approaches can fail to resonate with local actors. Four key patterns emerged: lack of contextual relevance, high administrative or procedural burden, detachment from local communication norms and overextension of too many selected value chains.

Generic online trainings delivered in English were frequently cited as a poor fit for many rural regions: *“generic workshops in English didn’t attract participants due to language barriers and the oversaturation of similar EU project offerings.” (H1)* Stakeholders described these sessions as too broad, abstract, or disconnected from their immediate needs. In some cases, language barriers made participation difficult, while in others, the virtual format failed to foster any sense of interaction or local relevance. Without hands-on examples or direct applicability, such trainings were often perceived as a ‘tick-the-box’ activity rather than a meaningful learning opportunity.

Actions perceived as administratively burdensome or overly formalistic (e.g. questionnaires) also undermined engagement. When stakeholders were required to navigate complex procedures or complete extensive documentation, without a clear benefit or support, enthusiasm quickly dissolved. Project activities that focused more on reporting requirements than on genuine co-creation were viewed as disconnected from stakeholder realities.

Top-down communication approaches proved ineffective in regions where trust in external institutions is low. Messaging delivered in institutional jargon or by unfamiliar actors was often met with scepticism or indifference. In such contexts, the identity of the messenger - and their ability to speak the ‘local language’, both literally and culturally - mattered as much as the content itself. Without locally embedded facilitators or credible intermediaries, even well-designed interventions struggled to gain traction.

Overextension across too many value chains or activity areas also emerged as a limiting factor. Stakeholders noted that when initiatives attempted to address too many topics or sectors simultaneously, efforts became diluted and outcomes less tangible: *“As a lesson learned, I would say that having three value chains is quite a lot. Maybe it would have been better to narrow it down, but in the end, we are really focusing on one value chain where the region is really involved.”* (H7) Instead, it was considered more effective to focus on a smaller number of high-potential, actionable areas where visible progress could be made. Narrowing the scope not only improved clarity but also increased the chances of delivering demonstrable results and building momentum for future engagement.

In summary, local actors respond best to actions that provide clear, concrete benefits, practical learning opportunities, and direct personal interaction with clear focus on few value chains. Effective hub establishment relies on targeted, collaborative events like study visits and B2B matchmaking, while avoiding generic online activities and administrative complexity. The most successful approaches are tailored to local needs and locally adapted value chains, leverage existing networks, and demonstrate tangible benefit to participants.

### Systemic conditions Facilitating and Hindering the Translation of Co-Creation into Practice in Regional Bioeconomy Hubs (RQ2)

The successful implementation of co-creation in regional bioeconomy hubs depends not only on the design of individual activities but on a broader set of systemic conditions. These enabling factors operate at the institutional, relational, and contextual levels and create the groundwork upon which specific engagement strategies can be meaningfully executed. Across regions participating in the RuralBioUp project, several common enablers were identified as critical to creating an environment in which co-creation could thrive.

***Embeddedness in Local Contexts and Bottom-Up Framing***

A fundamental enabler is the alignment of co-creation efforts with local priorities, mentalities, and production realities. Co-creation processes must be grounded in an understanding of local culture, values, and economic structures to generate relevance and legitimacy. A bottom-up approach ensures that stakeholders recognize their own challenges, interests, and knowledge in the agenda-setting process, rather than perceiving it as an externally imposed or top-down initiative. This contextual sensitivity is not merely a design choice. It is a precondition for authentic collaboration.

**Trust-Based Relationships and Network Capital**

The strength of existing local networks and relationships of trust significantly affects the feasibility of co-creation. Hubs that could build on pre-established platforms, such as farmer associations, local innovation intermediaries, or municipal networks, were more successful in securing engagement. These networks serve as social infrastructure for knowledge exchange and help reduce the perceived risk of participation. In several regions, ‘hub contact points’ (trusted individuals or organisations already embedded in the community) were instrumental in enabling dialogue and reducing scepticism: *“A key strategy is maintaining good working relationships. If people trust you and your work, they'll participate. The most challenging part is targeting people outside your existing network and keeping them engaged.”* (H7)

**Local Facilitation and Proximity of Coordination**

The presence of a dedicated local facilitator or contact person (someone who understands the regional context and is seen as part of the community) proved to be a major success factor. Unlike external experts, local facilitators can act as boundary spanners, translating between policy language and everyday concerns. Their proximity allows for informal, flexible, and responsive engagement, which in turn builds credibility and continuity in stakeholder relationships.

**Governance Flexibility and Institutional Support**

Co-creation is inherently iterative and adaptive. As such, successful implementation depends on institutional flexibility - the ability of projects and governing structures to adapt to stakeholder input, adjust timelines, and recalibrate strategies as needs evolve. Rigid governance mechanisms, in contrast, undermine co-creation by treating engagement as a checkbox activity rather than a dynamic, learning-oriented process. Supportive institutions allow co-creation processes to remain open-ended, participatory, and sensitive to changing circumstances.

**Visibility of Value and Incentive Alignment**

While co-creation is based on collective processes, individual stakeholders must also see personal or professional value in participating: *“They needed to see a ‘real impact or real change’ and know ‘what value you're bringing to their lives”* (H7). Hubs that clearly communicated potential benefits (such as access to funding, recognition, or new market opportunities) - created stronger incentives for engagement. When stakeholders understand how co-creation contributes to their own goals, they are more likely to invest time and effort in collaborative processes. H6 explicitly concretized that *"Concrete opportunities matter more than just networking”* (H6) and that stakeholders often sought funding opportunities (H6, H3)

***Structural Barriers Hindering Co-Creation in Practice***

While co-creation holds strong conceptual appeal and is widely promoted in policy frameworks, its implementation in rural bioeconomy contexts is often hindered by systemic constraints. These barriers are not simply logistical challenges. They reflect deeper structural issues, including institutional inertia, relational deficits, and misaligned incentives. Insights from the RuralBioUp project reveal how these barriers manifest in practice and how they can undermine the co-creative potential of regional bioeconomy hubs.

**Time Scarcity and Capacity Constraints**

One of the most consistent obstacles to co-creation is the limited availability and capacity of stakeholders, particularly those from the private sector such as farmers and small business owners: *"It is very hard to involve the private sector, especially farmers, because they had a ‘I don't have so much time, I don't want to lose time, maybe we'll see later’ mentality.”* (H7) Many operate with minimal surplus time or resources and are therefore unable to engage in ongoing, iterative processes without clear and immediate benefit. This scarcity of time restricts both the breadth and depth of stakeholder involvement, and makes co-creation vulnerable to becoming extractive, relying on occasional inputs rather than sustained collaboration.

**Stakeholder Scepticism and Cultural Resistance to Change**

In several regions, co-creation efforts were hampered by cultural conservatism and scepticism toward externally initiated innovation. Stakeholders, particularly in more traditional rural areas, were cautious about engaging with new initiatives unless they could see concrete, long-term value for their own enterprises or communities. In some cases, scepticism was rooted in past experiences with short-lived or top-down projects. Without deliberate trust-building and visible impact, co-creation risks being dismissed as another transient initiative disconnected from real-world concerns.

**Ambiguity Around Purpose and Value Proposition**

Hub facilitators reported, that a lack of clarity regarding the mission, scope, and added value of the bioeconomy hub was another significant barrier to engagement. Stakeholders often struggled to distinguish the hub from other existing clusters or support platforms, especially when communication was vague or overly technical. This conceptual ambiguity diminished perceived relevance and led to confusion or disengagement. Effective co-creation requires a clearly communicated, shared understanding of purpose. It must be established early in the process.

**Geographical and Infrastructural Limitations**

In large or sparsely populated regions, geographical distance and limited transport infrastructure made in-person collaboration difficult to sustain. While digital tools were employed to bridge these gaps, online engagement formats, particularly when conducted in English or lacking interactivity, often failed to attract meaningful participation. The digital divide, language barriers, and format fatigue contributed to a sense of disconnection from the process and reduced opportunities for trust-based relationship building.

**Institutional Rigidity and Power Asymmetries**

Co-creation thrives in flexible and inclusive governance environments. However, some hubs struggled with institutional rigidity that limited the responsiveness of project structures. Predefined deliverables, strict timelines, and procedural constraints left little room for adapting to stakeholder input or shifting priorities. Additionally, power asymmetries between different stakeholder groups sometimes led to imbalances in decision-making, where co-creation became symbolic rather than substantive. When stakeholders feel that decisions are made elsewhere, engagement dissolves and legitimacy erode.

### Enabling bioeconomy adoption in traditional rural areas (RQ3)

Introducing bioeconomy and innovation in traditionally conservative rural regions requires a cultural and psychological shift. Resistance often stems from deep-rooted worldviews and past experiences, not disinterest, therefore the top-down approaches tend to typically fail. Instead, having successful strategies in place helps to build trust, relevance, and co-adaptation, integrating new ideas into familiar contexts. Drawing on insights from the RuralBioUp project, mindset change demands patience, humility, and sustained engagement.

***Key Strategies for Enabling Innovation Through Co-Adaptation***

Building trust and awareness is a key strategy for conservative rural contexts. Making new approaches more relatable, acceptable, and contextually grounded following strategies were identified as facilitators for implementation:

**Co-creation grounded in local realities**

Rather than introducing fully formed solutions, successful initiatives engage stakeholders in co-creating innovations that adapt to local strengths and opportunities, assets, and addresses challenges. H5 explicitly stated the importance of *"… understanding the local context … on implementing a proposed action, it is crucial to consider the unique local context, including environmental, economic, and social factors that will influence the project's success.”* (H5) This co-creative process not only improves solution fit but also contributes to building legitimacy and reducing resistance by the engaged stakeholders.

As mentioned in the previous result, effective co-creation (also in conservative regions) involves starting with what works: identifying existing local practices, community structures, or resource flows that already align with bioeconomy goals, and then working collaboratively to enhance them. This fosters continuity, builds trust and commitment and embeds in existing environment, rather than asking stakeholders to abandon familiar systems. *“Younger stakeholders might be more receptive to online meetings, while older farmers or traditional stakeholders require face-to-face, traditional approaches. Working with farmer associations is crucial because they represent local culture and can help disseminate information.”* (H7)

Across all findings, one principle is consistently validated: innovation must be co-adaptive rather than disruptive. Co-adaptation implies a mutual adjustment process. New ideas are introduced in ways that are sensitive to local norms, while communities gradually adapt their mental models and practices. This approach is especially in traditional settings an important key factor to success. By embedding change within familiar reference points, using trusted messengers, and allowing time for iterative learning, co-adaptation ensures that the transition to a bioeconomy succeeds.

**Practical demonstrations and Peer-to-Peer learning**

One of the most effective strategies for mindset shift involves hands-on, visible examples that make abstract concepts tangible like the study visits. These practical demonstrations enable stakeholders to see innovations in action. Of particular importance is the use of peer-to-peer learning formats, often described as ‘farmers train farmers’ or ‘entrepreneurs train entrepreneurs.’ These approaches rely on trusted local actors to model change, reducing perceived risk and reinforcing legitimacy through social proof. Stakeholders are more likely to adopt new practices when they see them working for others in similar circumstances, rather than hearing about them from distant experts.

**Connecting innovation to familiar practices**

Innovation adoption improves when it is framed in relation to existing or traditional practices, rather than presented as entirely novel. H2 learned to *"… align with their interests and stay as close as possible to their local priorities"* because imposing something " from the ‘top down’ would lead to non-participation. It “… *has to be built bottom-up."* (H2) For instance, composting a core component of many bioeconomy systems can be accepted more readily when framed as an extension of traditional organic farming or waste reuse methods. This strategy supports the feeling that the innovation feels less disruptive and more like an evolution of known methods. According to the hub facilitators it also helps overcome the ‘foreignness’ of bioeconomy language and concepts. By localizing terminology and linking new practices to culturally resonant ideas, bioeconomy hubs can lower entry barriers and foster a sense of familiarity and ownership.

***Strategic Engagement and Incentives***

Strategic engagement and tailored incentives are essential for motivating participation in conservative rural contexts. Approaches that allow early engagement, demonstrating tangible benefits were identified as key facilitators for successful implementation.

**Early engagement of key stakeholders**

H3 discovered that direct, personal, face-to-face engagement was crucial for building trust and attracting participation, as emails and online methods were largely ineffective for the *"old fashioned thinking people"* (H3) in their region. Another essential element is the strategic involvement of key stakeholders, concretely respected local figures or multipliers, such as farmers’ association leaders, teachers, or regional development representatives. Their endorsement can significantly accelerate acceptance of new ideas, especially in regions where hierarchical or communal authority carries weight. Including younger generations - as both users and communicators of innovation - can also act as a social bridge between traditional mindsets and forward-looking practices.

**Demonstrating Tangible, Local Benefits**

Finally, successful strategies ensure that the benefits of innovation are visible, relatable, and locally adapted. Stakeholders are more likely to embrace new practices when they can observe positive outcomes, such as increased income, reduced waste, or improved crop resilience, within their own community. Innovation that delivers concrete, short-term value, even in modest ways, helps build the confidence necessary for longer-term transformation.

***Strategies overcoming barriers to mindset change***

Even with the right strategies, the process of shifting mindsets in traditionally structured rural environments faces persistent and deeply rooted challenges. These include risk aversion, economic uncertainty, and emotional attachment to established practices. Addressing these barriers requires not only effective engagement strategies, but also long-term commitment, cultural sensitivity, and strategic use of information to build both trust and confidence in new directions.

**Patience and Vision**

According to the hub facilitators, the most important factor in overcoming resistance is the recognition that mindset change is a gradual process. *“… they did not expect a large immediate effect from the RuralBioUp project, understanding that results would develop over time.”* (H2). In conservative regions, stakeholders (particularly small-scale farmers and entrepreneurs) often hesitate to experiment with new business models or value chains unless they see guaranteed returns. This caution is rational given the risks they face and the limited margins for failure.

As such, impatience or pressure for quick results can be counterproductive. Instead, projects must cultivate a shared vision for change and maintain consistent communication over time, even when immediate engagement is low. This includes listening carefully to local concerns, setting realistic expectations, and being visibly present in the region. Trust is built incrementally, and only once it is established can more transformative conversations take place.

**Data-Driven Decision Making**

Another effective way to overcome scepticism is through the use of robust data and evidence-based planning. When potential innovations (such as new value chains, biomass uses, or waste-to-resource strategies) are backed by regional studies, economic feasibility analyses, and sectoral data, stakeholders are more likely to consider them legitimate and viable.

Presenting data in an accessible and context-specific way – like provided by the RuralSpot - helps ground the discussion in logic. For example, desk studies that highlight available regional feedstocks or map existing supply-demand gaps make it easier for stakeholders to see where opportunities exist and how they might be leveraged. This reduces perceived risk and enhances the credibility of proposed changes.

**A Strategic, Culturally Sensitive Approach**

Ultimately, overcoming barriers to innovation in conservative rural environments is not about persuasion alone. It is about guiding a community through its own process of change. H2 experienced that their region, influenced by its history, leads people to be " *rather shy and conservative"* (H2) when it comes to new business ventures or startups, often needing guaranteed returns before investing (H2). This requires a nuanced strategy that balances empathy with leadership, and long-term vision with short-term wins.

By combining patience, relevance, and transparency, change agents can shift conversations from “Why change?” to “How do we make it work for us?” Through practical demonstration, peer-led engagement, and locally grounded co-creation, stakeholders begin to see innovation not as an external threat, but as a meaningful opportunity to shape the future of their region.

## Discussion

This study makes a novel contribution to the literature on regional bioeconomy hubs by extending the evidence from earlier initiatives such as BIOVoices ^1^ and BioBridges ^2^. While those projects largely emphasized knowledge sharing and policy dialogue, RuralBioUp demonstrates concrete co creation strategies that enable long term stakeholder ownership, even in conservative rural contexts. By combining empirical evidence from nine regional hubs with a focus on co adaptive approaches, this work fills a critical gap in the bioeconomy hub literature.

In doing so, it highlights the interplay between stakeholder perceptions of impactful actions, co creation practices, and mindset shifts in shaping the successful establishment and operation of regional bioeconomy hubs. The findings underscore how nuanced social and institutional dynamics influence the translation of bioeconomy concepts into sustainable, locally embedded innovations. This advances both academic understanding and practical knowledge on how to design context sensitive, trust based interventions that support rural transitions toward sustainability.

### Linking perceived effectiveness to implementation outcomes

Our results demonstrate a strong interplay between the perceived effectiveness of actions and actual implementation outcomes. Consistent with innovation systems and transition theory ^22, 34^, stakeholders’ engagement hinges not solely on the inherent quality or innovativeness of an intervention but on its relevance, legitimacy, and sense of ownership. Actions that resonate with local economic and social realities, are co-endorsed by trusted local actors, and allow stakeholders to see themselves reflected in the process (e.g., through co-design or public recognition) foster deeper commitment and sustained involvement. This aligns with findings in participatory governance literature emphasizing that legitimacy and local ownership are fundamental for sustaining innovation uptake ^28^.

Conversely, activities perceived as generic, administratively complex, or disconnected from immediate local interests tend to generate scepticism, disengagement, and may even damage trust in bioeconomy initiatives. This supports previous research warning against “one-size-fits-all” approaches and highlights the importance of tailoring strategies to local contexts ^58^. In practical terms, our findings emphasize that bioeconomy hubs must design engagement formats with stakeholders as co-creators rather than passive recipients, emphasizing tangible benefits such as learning opportunities, visibility, and funding access. This reinforces the call for stakeholder-centric models like co-creation for sustainability transitions, moving beyond tokenism toward genuine co-ownership.

### Institutional and relational preconditions for co-creation

The study further elucidates the complex conditions required to translate co-creation theory into effective practice. Consistent with broader literature on innovation ecosystems ^34^, co-creation is not merely a toolkit of participatory methods but demands an enabling governance environment characterized by institutional flexibility, equity, and sustained commitment. The identified structural barriers, such as stakeholder time constraints, scepticism rooted in conservative attitudes, and logistical challenges, highlight the persistent institutional inertia and trust deficits that hamper collaborative processes.

These findings stress the need for relational trust-building and transparent, stakeholder-centred communication as cornerstones of co-creation efforts. The governance arrangements must allow for adaptive management, recognizing and responding to local power imbalances and socio-cultural specificities. This contextual sensitivity is critical to fostering an inclusive innovation culture where diverse actors feel valued and motivated to contribute. In this respect, our results contribute empirical nuance to calls within transition studies advocating for governance approaches that bridge institutional frameworks and grassroots realities ^22^.

### Mindset shifts as a foundation for rural bioeconomy adoption

Finally, the research underlines that mindset change in conservative rural environments is a slow, iterative process rooted in trust, dialogue, and co-learning rather than sudden technological shifts or external interventions. This resonates strongly with transition literature emphasizing the socio-cultural dimension of systemic change ^22^. Bioeconomy adoption emerges when innovations are framed not as threats but as extensions or modernizations of existing local practices, legitimized through peer learning and visible local benefits.

The emphasis on co-adaptation over disruption highlights the importance of embedding innovation within familiar identities and narratives, facilitating acceptance by aligning with community values and reducing perceived risks. This process requires patience and long-term vision, recognizing that economic rationales alone cannot drive transformation in contexts where social cohesion and tradition are paramount. Our findings suggest that regional bioeconomy hubs can function as critical “bridges” that mediate between tradition and transformation, fostering sustainable development ^59^ pathways that are both rooted in local realities and oriented toward future resilience.

### Implications for policy and practice

The findings of this study highlight that implementing bioeconomy hubs in rural regions is not solely a technical or administrative endeavour, but a socio-institutional process grounded in trust, local relevance, and community ownership. For both practitioners and policymakers, this has direct implications for how rural bioeconomy transitions should be designed, supported, and evaluated.

First, engagement must be rooted in practical, context-specific formats that demonstrate visible value to participants. Actions such as study visits, peer-led demonstrations, and co-organized events in local languages were consistently more effective than generic trainings or top-down communications. These formats foster inclusion and ownership by connecting innovation to familiar experiences and everyday concerns. In contrast, excessive administrative demands, unclear messaging, and attempts to address too many value chains diluted effectiveness and discouraged participation.

Second, sustainable co-creation requires supportive institutional and relational environments. Trust-building, local facilitation, and flexible governance are essential to adapt activities to local needs and ensure iterative feedback loops. The presence of embedded intermediaries proved critical, being in the position to navigate networks, build trust, and communicate clearly. Conversely, rigid structures and externally defined deliverables hindered meaningful engagement.

Third, successful innovation in traditionally conservative rural settings depends on culturally sensitive, co-adaptive approaches. Rather than promoting disruptive change, effective strategies build on existing knowledge, values, and relationships. Framing bio-based innovations as extensions of traditional practices, involving respected community members early, and demonstrating visible, place-based benefits (e.g. increased income, improved resource use) foster legitimacy and reduce resistance. Stakeholders responded most positively when participation aligned with their professional goals, offered recognition, or enabled access to opportunities.

Finally, fostering long-term mindset change requires patient, sustained engagement. Transformative processes in rural bioeconomy contexts are cumulative and relational, not linear. Policies should therefore move beyond one-size-fits-all models and instead support adaptive implementation pathways that allow for local experimentation, trust-building, and shared narratives of progress.

**Figure 2.**
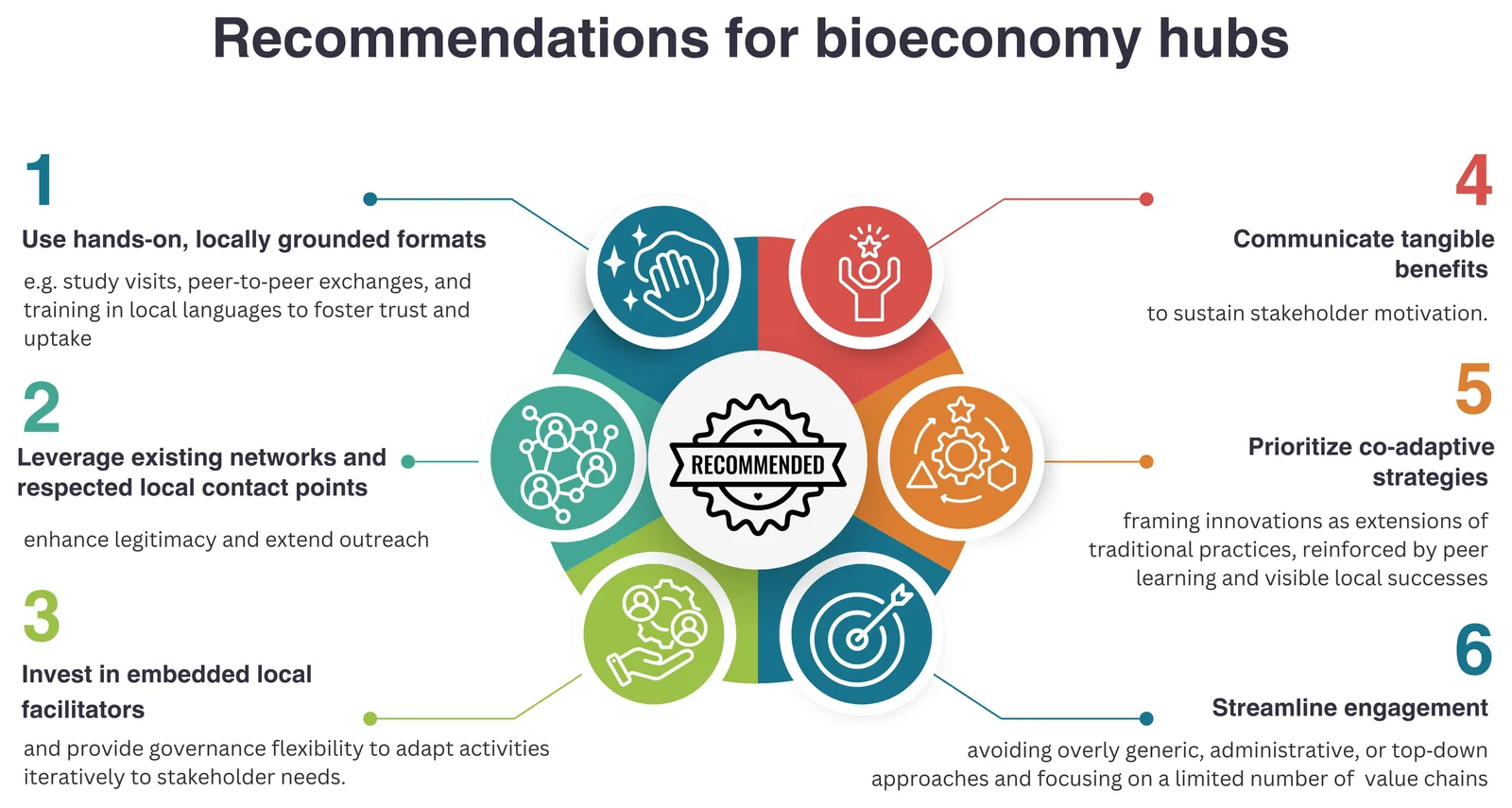
Overview Recommendations Hub Establishment. The figure summarizes six strategic recommendations derived from stakeholder experiences and analysis. These highlight practical approaches to foster trust, legitimacy, and sustained engagement, while ensuring hubs remain adaptive to local contexts and stakeholder needs.

Consequently, policy recommendations on a practical perspective ( Figure 2) tackle:

• **Use hands on, locally grounded formats** such as study visits, peer to peer exchanges, and training in local languages to foster trust and uptake.

• **Leverage existing networks and respected local contact points** to enhance legitimacy and extend outreach, especially in conservative rural settings.

• **Invest in embedded local facilitators** and provide governance flexibility to adapt activities iteratively to stakeholder needs.

• **Communicate tangible benefits** (e.g. funding access, recognition, business opportunities) to sustain stakeholder motivation.

• **Prioritize co adaptive strategies** that frame innovations as extensions of traditional practices, reinforced by peer learning and visible local successes.

• **Streamline engagement** by avoiding overly generic, administrative, or top down approaches and focusing on a limited number of high potential value chains.

By aligning bioeconomy initiatives with the lived realities of rural communities and fostering trust-based, co-adaptive engagement, policymakers can support bioeconomy hubs as effective vehicles for inclusive and sustainable change. The findings of this study therefore provide concrete entry points for EU, national, and regional strategies to translate the bioeconomy from vision into practice.

The results extend beyond the operationalization of individual hubs, offering direct implications for EU and national bioeconomy policy design. They highlight the importance of embedding co-creation and participatory governance principles into policy frameworks, ensuring that strategies are not only top-down but also responsive to diverse local contexts. By demonstrating how trust-building, peer-to-peer learning, and context-sensitive adaptation enhance uptake in conservative rural regions, the evidence supports policies that foster inclusiveness, legitimacy, and long-term sustainability.

At the EU level, the findings reinforce the objectives of the European Green Deal and the European Bioeconomy Strategy. Place-based and trust-driven approaches have proven more effective in securing long-term adoption, indicating that policies should promote flexible funding instruments that allow local adaptation and prioritize multi-stakeholder collaboration. Incorporating these lessons into forthcoming revisions of the EU Bioeconomy Strategy ^60^ and the Common Agricultural Policy (CAP) could strengthen alignment between high-level sustainability targets and grassroots implementation.

At the national level, the evidence underscores the need to institutionalize local facilitation roles and peer-to-peer learning mechanisms within bioeconomy strategies. Supporting the training and funding of embedded facilitators, incentivizing cross-sectoral networks, and reducing administrative complexity are crucial to increase legitimacy and participation. National strategies should avoid uniform, top-down models, instead enabling regional experimentation and long-term co-adaptive processes that build on local capacities and traditions.

Finally, the RuralBioUp findings stress the need for longitudinal evaluation beyond 2025 to assess the enduring impacts of co-creation-based hubs. Comparative analyses in non-EU and Global South rural contexts are also needed to test the transferability of these models under different socio-economic and institutional conditions. Together, these adjustments can transform bioeconomy hubs into powerful instruments for sustainable, inclusive, and climate-neutral rural development across Europe and beyond.

### Limitations

While this study provides valuable insights into the development and implementation of regional bioeconomy hubs, several limitations should be acknowledged.

First, the research is based on a qualitative, multi-case design involving nine hubs across Europe. While this allows for in-depth, context-sensitive analysis, the findings are not intended to support statistical data. Instead, they offer analytical generalization and transferable lessons that may inform similar initiatives in comparable contexts.

Second, the study relies heavily on self-reported data from hub facilitators, collected through interviews, workshops, and participatory sessions. This reliance introduces the potential for bias, as facilitators may emphasize certain successes or underreport challenges. To mitigate this, findings were triangulated with multiple data sources - including Mentimeter feedback, and workshop results -and validated by experts in two MML workshops. While this enhances validity, the interpretations remain subject to participant perception and selective memory.

Third, the project’s implementation timeframe (October 2022 – June 2025) may limit the ability to assess long-term impacts and sustainability of the hubs. The lessons learned reflect the hubs’ establishment and early operational phases rather than mature, fully embedded systems. Future research via mixed-methods research should therefore include longitudinal studies beyond 2025 as well as comparative analyses in non-EU rural contexts to assess long-term impacts and transferability.

Lastly, the diversity of regional and cultural contexts, while a strength for capturing variation, also introduces complexity in comparison. Some findings may be shaped more by national policy frameworks, institutional structures, or funding environments than by the specific strategies applied within the hubs themselves.

Despite these limitations, the study offers empirically grounded, practice-oriented insights that contribute to the growing body of knowledge on participatory bioeconomy development in rural regions.

## Conclusions

This study contributes to a deeper understanding of how regional bioeconomy hubs can be effectively implemented in traditionally conservative rural contexts by illuminating the perceived successful actions for implementation, co-creation dynamics, and mindset shifts. It underscores that the success of bioeconomy initiatives hinges less on technological novelty and more on perceived relevance, legitimacy, and stakeholder ownership. Actions that are practical, locally-based, and visibly beneficial were shown to strengthen engagement, while top-down or generic interventions often led to disengagement or resistance.

Importantly, the research reaffirms the need for co-creation that is not merely a participatory method but a governance approach that requires institutional flexibility, trust-based relationships, and cultural sensitivity. Regional bioeconomy hubs function most effectively when they act as bridges, connecting innovation with local identities, aligning new ideas with existing practices, and fostering long-term, trustful relationships between actors.

For policy and practice, the findings suggest that future bioeconomy strategies should prioritize targeted, tangible engagement formats; utilize local languages and networks; and create visible incentives for participation. Policy frameworks should support governance models that enable local flexibility, reduce administrative burdens, and fund local facilitation to sustain grassroots engagement. Embedding innovation through co-adaptation rather than disruption emerges as a key strategy for overcoming scepticism and fostering meaningful participation.

With appropriate support structures, iterative learning processes, and policy alignment, regional bioeconomy hubs like those in RuralBioUp can evolve into resilient innovation ecosystems. These hubs not only catalyse local transitions but also hold the potential to contribute meaningfully to broader European goals, advancing the bioeconomy in a way that is inclusive, locally grounded, and systemically transformative.

Still, future research should explore the long-term outcomes of such co-creative bioeconomy hubs, including their capacity to build local innovation ecosystems. In the same way, the exploration of the long-term structural change should become key topic for future research. Comparative studies across different governance settings and local differences could further clarify how institutional arrangements shape stakeholder engagement and innovation uptake. Additionally, more work is needed on the role and requested skills of intermediaries and facilitators in translating policy into practice, particularly in rural contexts where traditional values and risk aversion remain strong.

## Ethics and consent

All participants provided informed consent before taking part in the study. The process included a full briefing on the research purpose, use of contributions, data handling, and privacy protection. Participants were informed about the study objectives, the voluntary nature of their involvement, and their right to withdraw at any time without consequence. Consent was given with the explicit understanding that all data would be anonymized.

For interviews with hub representatives, participation has been agreed in the projects Consortium agreement. Nethertheless verbal consent was also obtained and audio-recorded at the beginning of each session. This approach was chosen to reduce administrative burden and to suit the conversational, interactive nature of the interviews. For the Mobilisation and Mutual Learning (MML) workshops, participants provided consent via an online registration form, and an additional consent sheet was available at sign-in to confirm participation on the day of the event. For hub exchange activities and the quantification of project phases, consent was covered under the project’s Consortium Agreement.

All participants were adults and provided their own consent. They were informed that they could withdraw their data at any stage by submitting a written request to the corresponding author. Quotations and findings are presented in a way that fully safeguards the identity of individuals and organizations. Ownership of co-created knowledge and data was managed in accordance with the Consortium Agreement, which defines rights and responsibilities for data use and dissemination.

## Data Availability

This study is based on qualitative data collected within Work Package 3 of the RuralBioUp project, covering seven countries: Italy, Romania, France, Ireland, Latvia, and the Czech Republic. The dataset consists of anonymized semi-structured interviews and workshop outputs. To protect participant confidentiality, all interview data were fully anonymized before analysis. Personal identifiers were removed or replaced with codes, and any context that might allow indirect identification was carefully reviewed. Metadata describing the dataset follows FAIR principles, ensuring that it can be discovered and reused under appropriate conditions. Access to the anonymized dataset can be requested by contacting the research coordinator, Margit Hofer ( hofer@zsi.at), at the Centre for Social Innovation (ZSI). Requests must include a written plan explaining the intended use of the data. Each request will be reviewed by the ZSI research coordinator in consultation with the RuralBioUp consortium to ensure compliance with ethical and legal requirements. Data ownership and conditions of reuse are governed by the RuralBioUp Consortium Agreement. Approved users will be required to comply with the project’s data management and confidentiality provisions. Further supporting materials, including interview guidelines and qualitative coding frameworks, are deposited in Zenodo: Centre for Social Innovation. (2025). *RuralBioUp qualitative data sets*. Zenodo. https://doi.org/10.5281/zenodo.17579049 ^61^ All data are available under the terms of the Creative Commons Attribution 4.0 International License (CC-BY 4.0). This section presents detail all novel data collected and used as part of our article. Details are hosted in the ZENODO repository from Margit Hofer, Martina Lindorfer and Samire Gurgurovci in 2025 under https://doi.org/10.5281/zenodo.17579049 ^61^ This project contains the following underlying data: Data file 1. (semi-structured interview questionnaire guideline) Data file 2. (7 anonymised interviews) Data file 3 (Graph materials for qualitative analysis) Data file 4 (Graph on recommendations) Data are available under the terms of Creative Commons Attribution 4.0 International Interview transcripts from the RuralBioUp projects were included in the data analysis.

## AI and generative AI use declaration

During the preparation of this manuscript, the authors used ChatGPT (version 4.0) for language editing and proofreading purposes. The AI tool was employed to review and refine the English language expression, grammar, and clarity of the written text while preserving the original meaning and scientific content. All substantive research content, analysis, interpretation of results, and conclusions presented in this work are entirely the authors' own. The use of this generative AI tool was limited to language enhancement and did not contribute to the conceptual framework, data collection, analysis, or interpretation of findings.
