# Unlocking regional bioeconomy transitions: Co-creation, stakeholder engagement, and systemic change in rural Europe

**DOI:** 10.12688/openreseurope.21341.2

**Published:** 2026-06-23

**Authors:** Margit Hofer, Martina Lindorfer, Samire Gurgurovci

**Affiliations:** 1 Zentrum für Soziale Innovation, Vienna, Vienna, 10150, Austria

**Keywords:** Bioeconomy hubs, Co-creation, Stakeholder engagement, Conservative rural contexts, Innovation adoption, Trust-based governance

## Abstract

**Background:**

Transitioning to a sustainable bioeconomy in rural Europe is challenged by the difficulty of translating high-level strategies into locally relevant initiatives. Regional bioeconomy hubs aim to bridge this gap, but their success hinges on engaging diverse, often conservative, stakeholders. While co-creation is a promising approach, its practical application in these contexts is poorly understood. This study examines the establishment of nine hubs within the Horizon Europe project RuralBioUp to identify effective strategies for fostering stakeholder ownership and systemic change.

**Methods:**

This qualitative study analysed multi-layered data collected from nine regional bioeconomy hubs between October 2022 and June 2025. Sources included seven semi-structured interviews with hub facilitators, two mutual learning workshops, participatory feedback tools, and co-created documentation. Thematic analysis identified patterns related to effective actions, co-creation dynamics, and innovation adoption.

**Results:**

Effective engagement actions were practical and locally grounded, such as study visits, targeted training in local languages, and co-organised events. Generic online workshops and bureaucratic activities proved ineffective. Co-creation was enabled by institutional flexibility, trusted local facilitators, and established networks. Key barriers included stakeholder time scarcity, logistical challenges, cultural scepticism, and unclear value propositions. In conservative settings, innovation adoption was facilitated through peer-to-peer learning and framing new ideas as extensions of existing practices.

**Conclusions:**

The success of regional bioeconomy hubs is contingent on trust, perceived local relevance, and stakeholder ownership, not just technological novelty. Instead of one-size-fits-all models, patient, bottom-up strategies that co-adapt innovation to community values are essential for long-term change. Hubs can act as critical facilitators for place-based transitions by embedding co-creation within flexible governance structures. Policy and practice should prioritize context-sensitive, tangible engagement to foster inclusive and sustainable rural bioeconomy development.

## Plain Language Summary

Across Europe, many rural areas are trying to move toward a more sustainable economy that makes use of local natural resources instead of relying on fossil fuels. This shift is called the bioeconomy. To support this change, the RuralBioUp project (2022–2025) created nine regional bioeconomy hubs in different European countries. These hubs are spaces where farmers, businesses, researchers, community groups, and local authorities can come together to share ideas and create solutions that fit their region.

Our study looks at what worked well and what did not, when setting up and running these hubs. We found that people were most willing to participate when activities felt useful, practical, and close to their daily lives. For example, visiting real-life bioeconomy projects, holding training sessions in local languages, and organizing events with local partners made a big difference. In contrast, generic online workshops in English or activities with heavy paperwork often discouraged participation.

Trust and personal connections proved essential. Hubs led by local facilitators who knew the community built more credibility and involvement than those relying on outside experts. Success also depended on showing clear benefits, such as new funding opportunities for bioeconomy initiatives, recognition, or business contacts.

Introducing innovation in traditional rural areas takes time. People are more open to new ideas when they see them as a natural extension of what they already know, rather than as something completely new. Peer-to-peer learning for example, farmers learning from other farmers, was especially effective.

Overall, we conclude that bioeconomy hubs can become engines of sustainable rural development if they are locally grounded, trust-based, and adapted to community values and needs. Instead of one-size-fits-all approaches, patient, bottom-up strategies are key to long-lasting change.

## Introduction

As Europe transitions towards a sustainable, circular, and inclusive bioeconomy, rural regions are widely recognised in the literature as playing a pivotal role in embedding bio-based solutions within local contexts. However, translating high-level strategies into actionable and regionally relevant initiatives remains a complex challenge.

The RuralBioUp project, funded under the Horizon Europe Framework, was developed to address this challenge. It supports the establishment and implementation of nine regional bioeconomy hubs across Europe. Bioeconomy hubs are physical or virtual platforms designed to facilitate collaboration among diverse stakeholders—such as entrepreneurs, researchers, policymakers, and local communities—towards the co-creation, innovation, and implementation of sustainable bioeconomy solutions. These hubs served as focal points for developing region-specific bioeconomy initiatives that integrate local resources, expertise, and socio-economic contexts.

Each hub operated in a unique environmental context. They selected two value chains and tailored their actions to local needs and opportunities. By fostering multi-stakeholder collaboration through co-creation, knowledge exchange, capacity building, and integrating bioeconomy into regional development, the hubs contributed to creating a more resilient and locally adapted bioeconomy.

Despite increasing policy attention on the bioeconomy as a driver of sustainable regional development, practical implementation of bioeconomy hubs remains challenging. Previous research suggests that success is closely linked to the active involvement of and co-creation by diverse and often traditional local stakeholders. These include public authorities, SMEs, farmers, regional developers, civil society, and knowledge institutions. Co-creation has been identified in the literature as a promising approach to fostering inclusive engagement and developing context-specific solutions. Consequently, co-creation refers to a collaborative process observed in several bioeconomy hubs, where a diverse group of stakeholders actively engage in the design, development, and implementation of bioeconomy solutions. By fostering mutual learning and shared ownership, co-creation ensures that the resulting solutions are both locally relevant and context-sensitive, reflecting the specific needs and values of the communities involved. Yet, little is known about how co-creation processes operate in practice within regional bioeconomy settings. Questions remain about which conditions enable or hinder stakeholder participation, and which strategies effectively overcome common barriers.

This work provides empirical insights into how bioeconomy can be realised at the grassroots level. It also aligns with broader European objectives, such as the Green Deal, rural development, and the transition to climate-neutral societies.

It explores which actions are perceived as effective, how co-creation supports the establishment of regional bioeconomy hubs, and how implementation is perceived to succeed in traditional settings. The study presents key lessons from the implementation of these hubs conducted between October 2022 and June 2025.

Using a multi-layered qualitative research approach - including semi-structured interviews, two mutual learning workshop outputs, co-created documentation, and participatory feedback tools - the study identifies perceived patterns of success, persistent challenges, and transferable practices in rural bioeconomy development.

Research Questions

The study addresses three research questions:

• RQ1: Which actions are perceived by local facilitators as effective or ineffective in establishing regional bioeconomy hubs?
• RQ2: What systemic and contextual conditions facilitate or hinder the practical implementation of co-creation in developing regional bioeconomy hubs?
• RQ3: What strategies have proven effective in initiating mindset shifts and enabling the adoption of bioeconomy and innovation in traditionally structured or conservative rural environments?

Through this work, we seek to contribute to the growing understanding of how bioeconomy can be realised at the grassroots level. We offer a nuanced view of how collaborative innovation processes can be operationalised in regional bioeconomy contexts.

Our findings build on evidence from earlier initiatives such as BIOVoices ^1^ and BioBridges. ^2^ While those projects mainly focused on knowledge sharing and policy dialogue, RuralBioUp extends this work by providing empirical examples of co-creation strategies that are associated with long-term stakeholder ownership, even in conservative rural settings.

The novelty of this study lies in combining empirical evidence from nine regional hubs with a focus on co-adaptive approaches. In doing so, it addresses an identified gap in the bioeconomy hub literature.

The findings are intended to inform both future research and practical efforts. They provide insights into strategies for designing effective stakeholder engagement and implementation frameworks for sustainable bioeconomy initiatives. These insights are relevant in the context of broader European goals such as the Green Deal, rural revitalisation, and the transition to climate-neutral societies.

This paper therefore outlines the theoretical framework and the RuralBioUp hub concept. It then describes the research methodology, presents the key findings from the multi-layer qualitative analysis, and discusses their implications for regional bioeconomy development. Finally, it concludes with broader lessons and future directions for policy and practice.

## Conceptual framework

The transition towards a sustainable and circular bioeconomy in Europe is widely understood to involve more than technological innovation. It is also associated with profound social, institutional, and cultural change, particularly in rural regions. High-level strategies and funding mechanisms exist to promote bio-based solutions. However, their effective implementation on the ground is often described as being closely linked to local engagement, institutional readiness, and socio-cultural contexts. The RuralBioUp project, through the establishment of nine regional bioeconomy hubs, provides a valuable perspective into real-world processes. It demonstrates how bioeconomy innovation can be initiated and sustained in diverse rural settings.

This chapter lays the theoretical foundation for analysing the three central research questions of the study. First, it examines how local actors perceive specific actions either as effective or ineffective in establishing regional bioeconomy hubs (RQ1). Second, it investigates the conditions - both facilitators and barriers - that shape the translation of co-creation theory into practice (RQ2). Third, it explores the strategies that have been reported as effective in introducing bioeconomy and innovation in traditionally structured or conservative environments. In such contexts, resistance to change and established mindsets can hinder the adoption of innovation (RQ3). To address these questions, the conceptual framework draws on interdisciplinary literature. It integrates insights from rural development, innovation studies, transition theory, and co-creation approaches. By combining these perspectives, the chapter highlights the interplay between structural conditions, cultural norms, and institutional environments, which shapes how bioeconomy implementation unfolds in practice. The goal is to identify where empirical findings from the RuralBioUp project can enrich scholarly understanding. In particular, the study seeks to contribute to knowledge on participatory innovation and system change in rural contexts.

### Bioeconomy in local/regional contexts

Bioeconomy in rural settings can be understood as a place-based innovation strategy that builds on the unique assets, knowledge, and capacities of specific regions to drive sustainable economic development. ^3^ ^,^ ^4^ Rather than applying uniform solutions, approaches need to be adapted or tailored to each region’s ecological, economic, and socio-cultural context. ^5^

Thus, innovation is closely tied to the local context by recognising the availability of natural resources in the region. It also considers the potential of cultural practices, institutional arrangements, and existing stakeholder networks. Recent empirical work underscores *“… the importance of participative approaches as key to the development of bioeconomy strategies, for which a key issue is the active engagement of a great diversity of stakeholders and the establishment of an inter-and cross-sectoral, as well as inter- and transdisciplinary cooperation in the region”.* ^6^ ^,^ ^7^

In addition, spatially sensitive approaches have been described in literature as important, as they help to ensure that innovation strategies respond to the territorial and ecological characteristics of rural regions. ^6^ ^,^ ^8^ This suggests that successful bioeconomy transitions often involve multiple dimensions and perspectives and are associated with interdependencies of regional embeddedness that can foster social sustainability and resilience. ^5^ ^,^ ^6^ ^,^ ^9^ ^,^ ^10^ Thus, bioeconomy has evolved beyond traditional pathways of production based solely on sustainable materials, increasingly encompassing broader societal transformations toward sustainable development along the entire value chain. ^11^ ^,^ ^12^ This also highlights the role of guidance and coordination in supporting stakeholders such as farmers, local developers, researchers, policymakers, government, and business in navigating pathways toward sustainable innovation.

Accordingly, a holistic and multidisciplinary perspective has been emphasised in the literature as necessary to address the complexity of circular bioeconomy across the entire value chain and its diverse stakeholders. This complexity arises from the interplay of multiple sectors, fragmented governance structures, potentially conflicting policy objectives, regional differences, and the need to integrate environmental, economic, and social sustainability considerations. ^13^ ^,^ ^14^

Consequently, the reviewed literature provides robust theoretical and empirical grounding for the argument that rural bioeconomy hubs can be conceptualised as place-based, context-aware initiatives. Rooted in co-creation and participatory governance, they can be associated with the potential to contribute to locally relevant, resilient, and sustainable outcomes.

### Bioeconomy hubs as incubators for systemic change and local adaptation

Fostering this systemic shift, the practical implementation has been associated with the emergence and use of bioeconomy hubs. ^15^ Bioeconomy hubs have been described as facilitators of systemic change and local adaptation by catalysing multi-stakeholder collaboration. They align innovation to regional contexts and embed circular bioeconomy solutions within existing regional socio-ecological potentials and opportunities. ^6^ ^,^ ^15^ ^,^ ^16^

These hubs can act as innovation-incubators that aim to orchestrate regional stakeholders’ needs and requirements, such as primary producers, regional developers, public authorities, SMEs, research institutions, and civil society. Often the value chains draw on the (local) knowledge of these stakeholders to adapt to local biomass availability, infrastructure, and policy conditions. ^17^ In this regards, empirical studies demonstrate that hubs build dynamic capabilities. ^10^ These include the abilities to sense, seize, and adapt towards a knowledge-based bioeconomy.

Other case studies emphasise how district-level bioeconomy strategies (created through stakeholder workshops and quadruple-helix governance) are operationalised locally which are associated with context-sensitive implementation. ^10^ ^,^ ^16^

Moreover, the concept of systemic innovation, characterised by cross-sector stakeholder integration and collaboration, is described in literature as an important mechanism for operationalising the bioeconomy across value chains. It is associated with enabling coordinated transitions that align economic, environmental, and social objectives ^17^ ^,^ ^18^ .

Circular bioeconomy in particular has been described as a driver of system integration, bringing together agriculture, forestry, aquaculture, and innovation into cohesive regional systems. ^19^

From a governance perspective, hubs can be understood as intermediaries that create connections, reduce coordination failures, and accelerate transitions. This perspective links bioeconomy hubs to broader transition studies and intermediary typologies. Likewise, the notion of hubs as living labs resonates with the literature on participatory and experimental innovation environments, ^20^ which emphasises their role as structured spaces for testing, co-creation, and diffusion.

Collectively, the reviewed literature suggests that bioeconomy hubs can be understood as more than demonstration centres. They are described as local connectors that may support and contribute to local transitions and reinforce regional resilience, adaptability, and sustainable innovation.

In line with the existing literature, the nine RuralBioUp hubs defined the hubs as “ *… collaborative space and facilitation tool that brings together diverse stakeholders - such as entrepreneurs, researchers, innovators, policy makers, civil society, media, and other regional actors - across the entire bioeconomy value chain. Its purpose is to foster dialogue, knowledge exchange, and networking in order to identify synergies, share best practices, and co-create solutions to regional and cross-regional challenges and opportunities in the bioeconomy. Through training, consulting, and strategic collaboration, bioeconomy hubs aim to build capacity, influence policy, and support the effective and sustainable use of renewable biological resources. Ultimately, they contribute to the development of functioning bioeconomy systems that align with broader goals such as regional development, climate neutrality, and the European Green Deal. The implementation differs highly from the regional identified opportunities, chances and cultural working modes.*” (Co-created bioeconomy hub definition, RuralBioUp consortium, Brussels May 2025).

This definition of RuralBioUp hubs positions them as incubators for systemic change and local adaptation. By intentionally bringing together a broad spectrum of stakeholders across the bioeconomy value chain - including not only entrepreneurs and researchers but also civil society and media, the hub becomes a structured space for cross-sectoral experimentation and learning. ^18^ ^,^ ^20^ ^,^ ^21^ This is associated with fostering the emergence of new governance modes, business models, and innovation pathways. These can challenge and gradually replace traditional, linear economic systems. Moreover, the emphasis on dialogue, co-creation, and knowledge exchange indicates a commitment to adaptive, participatory processes. ^22^ These processes are discussed in the literature as important for managing the complexity of systemic transitions. The inclusion of regional variation - acknowledging that implementation “differs highly” based on local opportunities and cultural modes - further reinforces the hub’s function as a locally embedded, flexible mechanism, that is able to respond to unique socio-economic and environmental contexts. Training, consulting, and strategic collaboration can be interpreted as mechanisms not only for capacity building but also for embedding new practices and norms into regional systems. In this way, they are associated with contributing to both structural transformation and context-sensitive innovation. ^23^ ^,^ ^24^ Based on the reviewed literature, hubs can be conceptualised as dynamic interfaces and may serve as engines for place-based systemic change.

In addition to environmental and social sustainability, economic sustainability plays a pivotal role in ensuring the long-term viability of bioeconomy hubs. For hubs to be effective, they shall develop financially sustainable models that generate consistent revenue streams. These models may include market-driven mechanisms, public-private partnerships, or cooperative ownership structures that allow hubs to continue operations without relying solely on external funding. The focus on economic sustainability is associated with ensuring that bioeconomy hubs are not just short-term interventions but are capable of growing and evolving within their local contexts.

Furthermore, robustness - the ability of bioeconomy hubs to cope with setbacks and failures- is seen as a critical factor for long-term success. Hubs that integrate mechanisms for adaptation and failure recovery tend to be more resilient in the face of challenges. This can be achieved through peer-to-peer learning, shared-risk investment models, and feedback loops that allow hubs to adjust to changing conditions. In rural settings, where failure or resistance to innovation is common, creating a culture of safe experimentation enables stakeholders to test ideas without the fear of disproportionate loss, ultimately fostering innovation.

### Multi-stakeholder integration via co-creation in bioeconomy development

The demand for holistic strategies has led to increased attention to inclusive, participatory approaches. In this context, participatory methods like co-creation and multi-stakeholder engagement are often described as aligning with societal adaptation, local requirements, and sustainable implementation. ^25^

The term ‘multi-stakeholder’ refers to the intentional inclusion and engagement of diverse actor groups (such as public authorities, businesses, academia, civil society, and citizens) in collaborative processes of problem framing, decision-making, implementation, and evaluation. Multi-stakeholder approaches aim to integrate plural perspectives, build legitimacy, and enhance the relevance and acceptance of innovation and governance outcomes. ^22^ ^,^ ^26^ While inclusive and multi-stakeholder approaches are widely promoted in bioeconomy and transition literature (e.g. ^22^ ^,^ ^27^), their relevance is context-dependent. In this study, inclusiveness is not treated as an inherent normative requirement but as an empirically observed factor associated with higher perceived legitimacy, stakeholder engagement, and implementation feasibility in the analysed rural contexts. In the context of bioeconomy development, multi-stakeholder processes are considered particularly valuable for aligning innovation strategies with local needs and are associated with managing complexity across sectors and fostering systems thinking. ^28^ This approach is commonly linked to principles of participation and responsible research and innovation (RRI). RRI promotes anticipatory, reflexive, and inclusive approaches to innovation that align with societal values and address grand societal challenges. ^23^ ^,^ ^29^

Building on multi-stakeholder engagement, co-creation on the other hand can be understood as a structured approach through which diverse actors collaborate and jointly shape solutions via shared learning and iterative knowledge integration. It refers to a collaborative process in which actors jointly define problems and develop solutions through mutual learning and the integration of different forms of knowledge. While co-creation is often described in the literature as emphasising inclusiveness, reflexivity, and context sensitivity ^25^ ^,^ ^30^ the extent and form of stakeholder inclusion may vary depending on the specific context and phase of the innovation process. In sustainability and bioeconomy contexts, co-creation has been associated with the development of shared ownership and system-level learning, which in turn have been linked to more locally embedded innovation forms of innovation. ^31^ ^,^ ^32^ Rooted in innovation systems theory and transition theory e.g., ^18^ ^,^ ^33^ co-creation highlights the systemic nature of innovation, recognizing that knowledge is generated not only by experts but also through experiential, contextual, and tacit knowledge held by practitioners. In practice, stakeholders’ involvement may extend across multiple stages of innovation cycle, from agenda-setting and design to implementation and evaluation, although the degree of participation is not necessarily uniform throughout.

Thus, the involvement of multi-stakeholder groups is commonly described as an important component of co-creation approaches. The integration of a broad spectrum of societal actors has been argued to increase the likelihood that outputs are perceived as relevant across different stakeholder group. ^22^ At the same time, such approaches are increasingly reflected in EU bioeconomy policy frameworks, for example through the concepts of Responsible Research and Innovation (RRI) and Mobilisation and Mutual Learning (MML). These frameworks promote multi-actor engagement as a mechanism to align innovation processes with societal needs and sustainability objectives ^34^ although their practical implementation and effectiveness remain dependent on institutional and regional contexts. ^35^

While RRI promotes anticipatory, inclusive, and reflexive research and innovation processes that align with public values and address grand societal challenges, ^27^ MML emphasizes horizontal learning, trust-building, and co-production of knowledge among diverse stakeholders. These processes have been described as important for overcoming scepticism and fostering legitimacy in traditional or conservative contexts. Social learning is therefore considered as an important component, since it enables actors to collectively reflect, adapt, and embed new practices in governance systems. ^22^ Other bioeconomy hubs like BIOVoices ^1^ or BioBridges ^2^ which adopted this format to co-develop knowledge, policy recommendations, and innovation pathways exemplify this approach. They are described as facilitating stakeholder dialogue, aligning innovation strategies with societal values, and translating shared challenges into collaboratively designed action plans. ICLEI’s ^35^ implementation of MML further demonstrates how such processes can contribute to building trust, legitimacy, and shared policy ownership across regions. ^35^

Despite this promising approach, co-creation faces significant requirements and barriers, including trust deficits, limited motivation and imbalances among stakeholders. Trust has been identified in multiple studies as a foundational condition for meaningful engagement. A lack of trust can hinder true participatory processes. ^36^ ^,^ ^37^ Motivation is also seen as a central element of participatory approaches. Stakeholders are more likely to dedicate their limited time and energy when they perceive tangible relevance, benefits, and a genuine sense of ownership in the initiative. Equally important in participatory governance is the balanced distribution of knowledge, interests, and representation across stakeholders, which is considered necessary to prevent power imbalances. Design elements aimed at inclusion are described as important for reducing power imbalances that distort deliberation and marginalize vulnerable stakeholders. Unchecked disparities in instrumental, structural, or discursive power are shown to undermine collaboration and social learning in governance processes. In rural bioeconomy settings, power imbalances are common, as local actors often follow established hierarchies or lack the resources to engage equally with researchers or government officials. More broadly, sustainability transition scholars emphasize that shifts in power relations are central to enabling systemic change. ^32^

To overcome these challenges, bioeconomy hubs and regional facilitators may adopt deliberate strategies to support mindset change, build social capital, and enable peer-to-peer activities. Structured methods such as deliberative workshops, living labs, and stakeholder roundtables can create spaces where actors safely express concerns, negotiate priorities, and build shared visions. Systemic innovation driven by multi-actor collaboration and stakeholder alignment is described in the literature as an important mechanism for operationalising the bioeconomy across value chains, as it is associated with facilitating coordinated transitions that balance economic, environmental, and social objectives. ^38^ ^,^ ^39^

Ultimately, co-creation in the bioeconomy has been conceptualised in the literature as more than consultation. It is described as a transformative practice that can reshape how innovation is understood, governed, and implemented. When designed thoughtfully, multi-actor approaches are associated with fostering legitimacy, adaptability, and impact in regional bioeconomy transitions, particularly in traditional and risk-averse settings. ^40^

### Co-creation in relation to perceived effectiveness of actions

The implementation of regional bioeconomy hubs cannot be evaluated solely by technical design quality or alignment with high-level policy goals. Rather, the perceived effectiveness of actions - how they are interpreted, experienced, and valued by local stakeholders - is considered important for their uptake and sustainable implementation. Co-creation, by facilitating inclusive, place-based development, was observed in this study to be associated with enhanced perception of effectiveness and stakeholders ownership, particularly where stakeholders perceived relevance to their local context. ^25^ ^,^ ^40^

Recent literature stresses that co-creation processes involving multiple stakeholder groups improve both, the relevance and credibility of regional innovation strategies. ^30^ ^,^ ^31^ Through early and ongoing involvement in agenda setting, planning, and implementation, local actors can express their context-specific needs, expectations, and constraints. This embeddedness is associated with fostering interventions that stakeholders perceive as more meaningful, timely, and legitimate, even when conventional performance metrics such as the number of events or pilot projects might suggest only modest progress. ^30^ Importantly, perceived effectiveness is not merely a subjective opinion. It influences real behaviours, including the willingness of actors to collaborate, invest effort, and sustain initiatives beyond the funding period. ^32^ If actors view hub activities as aligned with their local reality and values, they are more likely to mobilize their networks and resources in supporting them.

Local ownership is described as a fundamental mechanism through which co-creation enhances both perceived and actual effectiveness. When local actors shape not just goals but also the design and delivery of hub activities, they gain a sense of agency and control over outcomes. This aligns with Arnstein’s classic notion of participation as empowerment through citizen control ^41^ and with more recent insights on collaborative governance emphasising shared authority and legitimacy. ^42^ ^,^ ^43^ Participatory approaches have been described in the literature as potentially contributing to empowerment and broader stakeholder inclusion. ^44^ ^,^ ^45^ This is linked to intrinsic motivation, strengthens emotional and cognitive investment, and increases the likelihood that new practices will be maintained over time. Ownership also has been associated with social capital. ^46^ This is particularly important in rural areas where trust, reciprocity, and informal networks play a significant role in mobilizing action. In such settings, accounting for stakeholder engagement while acknowledging and appreciating their fundamentally different judgements, value frames, and viewpoints is vital. ^44^ Co-created initiatives are more likely to reflect local narratives and leverage existing capacities, contributing to higher uptake and reduced friction.

Beyond initial engagement, co-creation processes that include local actors in monitoring, evaluation, and adjustment of hub activities are associated with strengthening trust and transparency. This dynamic feedback-driven governance supports adaptive implementation and allows for the refinement of goals and methods in response to evolving conditions or unforeseen barriers implementation. ^28^ For example, in the RuralBioUp project, regional co-creation hubs surfaced early barriers (via SWOT analysis) such as lack of bioeconomy knowledge, limited innovation mindset, or fragmented stakeholder interest. These insights enabled timely adaptations and the development of solutions that were locally relevant and thus more readily accepted.

In rural development literature, the context-specific nature of challenges - such as remoteness, limited infrastructure, or path-dependent institutional practices - has been described as making standardised models of implementation ineffective. ^47^

Co-creation can facilitate embedding in the local context, as hub strategies are continuously adapted to local realities through direct interaction with stakeholders. This adaptability is particularly relevant for bioeconomy hubs, which must navigate diverse environmental, economic, and social systems. Co-creation is associated with providing the flexibility required to respond to local constraints and opportunities. The process itself can contribute to perceived effectiveness, as stakeholders value not only the outcomes, but also the extent to which the process accommodates their input, adapts to change, and respects local knowledge and capacity.

In sum, the perceived effectiveness of regional bioeconomy hub implementation is not simply an outcome but can be understood as a product of how participatory implementation is conducted. Co-creation is associated with improving perceived effectiveness by enhancing the effectiveness of actions, supporting the building of local ownership and motivation, facilitates adaptation to local needs, and fosters trust, learning, and sustained commitment. Embedding bioeconomy hubs in co-created, participatory processes is therefore conceptualised in the literature as both a normative ideal and an empirically supported approach associated with increased effectiveness, resilience, and long-term viability. ^42^ ^,^ ^48^

### Innovation as a systemic shift in traditional and conservative settings

Innovation in rural and traditional settings is not merely a matter of introducing new technologies or practices. It is often described as involving a systemic shift that fosters transformation at institutional, cognitive, and social levels. ^14^ Rural areas often follow established paths shaped by historical routines and entrenched institutions, making it difficult to adopt new approaches. ^10^ Innovation is frequently characterised in the literature as less about disruption and more about navigating and reshaping these complex systems through agency, adaptive governance, and networked strategies. Such processes resonate with the multi-level perspective on socio-technical transitions, which highlights how innovations emerge in niches and gradually reshape existing regimes under broader landscape pressures. ^49^ ^,^ ^50^ Innovation has been characterised in literature as less about disruption and more about navigating and reshaping these complex systems through agency, adaptive governance, and networked strategies. ^40^

Empirical research ^47^ underscores that rural innovation often builds on strong local networks and social capital. It relies on incremental path development and interactive governance rather than radical technological change. Effective transformation in such contexts has been linked to bridging roles, such as network enablers and knowledge brokers, which support adaptive governance and transformative social innovation. Rural innovation is often conceptualised as requiring systemic approaches that respect historical path-dependencies, leverage community agencies, and operate within socio-technical regimes to realise sustainable and locally embedded outcomes. Grassroots innovations are particularly relevant here, as they highlight how communities can generate bottom-up solutions aligned with local values and practices. ^51^

From an economically driven approach, a systematic review of high-quality studies ^52^ identified four key dimensions that consistently drive the adoption of green innovation. They refer to government policies and regulations, technological capabilities (availability, affordability, and suitability), organisational culture and human resource (HR) practices, and market dynamics including consumer demand. Additional influences include economic incentives, environmental concerns, strategic orientation, firm characteristics, and perceived environmental risks. Overall, green innovation adoption is shaped by a dynamic interplay of institutional, technological, organisational, and market factors.

On a socio-economic level, the Diffusion of Innovations theory ^53^ offers a valuable lens to understand how innovation adoption unfolds within society. Rogers ^53^ defines diffusion as the process by which an innovation is communicated over time through specific channels within a social system. An innovation refers to an idea, practice, or object that is perceived as new by an individual or adoption unit. The diffusion process involves both mass media and interpersonal communication channels. Rogers identifies key adopter categories (from innovators and early adopters to experts) and emphasizes how factors such as perceived relative advantage, compatibility with existing values, and complexity influence the rate and success of adoption.

In traditional rural contexts, many stakeholders fall into the more cautious adopter categories. This has been associated with a general risk aversion, limited exposure ^54^ to external innovation networks, and reliance on established practices. These conditions can create resistance to change, particularly when innovations are perceived as externally imposed or disconnected from local priorities and experiences.

Addressing these challenges has been argued to involve targeted strategies such as mindset change, trust-building, and peer-to-peer learning. Mindset change is typically described as a gradual process, shaped by repeated exposure to innovation narratives, visible local success stories, and the gradual reduction of scepticism. Trust-building is widely identified as important, particularly in contexts where top-down initiatives have historically failed or created dependency. In this regard, bioeconomy hubs can be understood as playing an intermediary role, acting as facilitators that create safe spaces for experimentation, dialogue, and participatory co-creation, thereby aligning innovation processes with local values and lived realities.

Additionally, peer-to-peer learning has been identified as an effective mechanism for overcoming cognitive and cultural barriers to innovation adoption. When farmers, entrepreneurs, or community leaders observe their peers successfully implementing bio-based practices - such as circular farming models, cooperative biomass valorisation, or new forms of regional marketing - they are more likely to reconsider their own positions. Such horizontal learning is associated with building legitimacy and trust, especially when reinforced by existing social networks and (influential) local stakeholders who can act as innovation brokers within their communities. Empirical studies confirm that regions with strong social capital and embedded peer networks exhibit higher openness to rural innovation. ^46^ ^,^ ^54^

Thus, fostering innovation in traditional settings is not only associated with technical or economic introduction. In contexts characterised by economic constraints and high uncertainty, the ability to experiment without incurring disproportionate losses is considered an important enabling condition. ^55^ Mechanisms such as pilot projects, shared investment models, and institutional safety nets can reduce perceived risks and support incremental adoption pathways. ^56^ These considerations are particularly relevant in rural settings, where limited financial margins and strong path dependencies may otherwise constrain innovation uptake.

### Synthesis: framing the research questions

This study adopts an interdisciplinary framework combining innovation theory, co-creation, rural sociology, and bioeconomy implementation. It examines how regional bioeconomy hubs can be understood as systemic transformation in rural areas. Recognizing that the shift to a circular bioeconomy entails not merely technological but also institutional and socio-cultural change, the research positions bioeconomy hubs as place-based innovation infrastructures. These hubs are described as mobilising local resources, fostering cross-sectoral collaboration, and responding to region-specific needs. In this study, co-creation is treated as both a theoretical and practical approach to inclusive innovation. It is grounded in transition studies, innovation systems, and participatory governance. Co-creation is associated with enhancing perceived effectiveness, fostering local ownership, and increasing the legitimacy and sustainability of interventions. The study addresses an identified gap between policy aspirations and on-the-ground practices in rural bioeconomy development, particularly in conservative or traditionally structured regions. It investigates three core research questions:

• RQ1 explores stakeholder perceptions of what actions are effective or ineffective in establishing bioeconomy hubs, linking perceived effectiveness to engagement and implementation outcomes.
• RQ2 examines enabling and constraining conditions for translating co-creation theory into practice, identifying real-world challenges such as power imbalances, institutional inertia, and trust deficits.
• RQ3 investigates strategies for introducing innovation in traditional rural settings, with particular focus on adaptive approaches in the studies contexts, drawing on trusted networks, peer learning, and visible local benefits.

Through these questions, the study seeks to contribute to a deeper understanding of rural innovation processes, offering empirical insights for improving stakeholder engagement, hub implementation, and socially inclusive bioeconomy transitions. The findings are intended to inform both academic discourse and policy practice.

### The RuralBioUp hubs framework

The understanding of hubs can differ substantially in terms of goals, functions, aims, and objectives.

As for the RuralBioUp bioeconomy hubs, the partners of the project collaboratively defined the hubs as a collaborative space and facilitation tool. These hubs bring together diverse stakeholders - such as entrepreneurs, researchers, innovators, policymakers, civil society, media, and other regional actors - across the entire bioeconomy value chain. Their purpose is to foster dialogue, knowledge exchange, and networking. They aim to identify synergies, share best practices, and co-create solutions to regional and cross-regional challenges and opportunities in the bioeconomy. Each RuralBioUp hub is facilitated by a hub facilitator who coordinates and implements hub activities to support innovation and collaboration among stakeholders. They guide the process by fostering communication, managing resources, and ensuring effective participation throughout the innovation cycle. Through training, consulting, and strategic collaboration, bioeconomy hubs aim to build capacity, influence policy, and support the effective and sustainable use of renewable biological resources. Ultimately, they are described as contributing to the development of functioning bioeconomy systems that align with broader goals such as regional development, climate neutrality, and the European Green Deal. ^57^

## Methodology

The definition of the hubs frames the research field as a collaborative space and facilitation tool that brings together diverse stakeholders across the entire bioeconomy value chain. This co-created definition outlines the core objectives and functions of the hubs while also reflecting the approach for implementation and types of actions undertaken.

Given the diversity of regional settings, the evolving nature of bioeconomy initiatives, and the importance of capturing both successes and challenges in implementation, qualitative methods were considered appropriate for uncovering in-depth insights that would not be accessible through purely quantitative approaches. This mixed-method approach allowed us to understand not only what happened in each hub, but also how and why certain dynamics unfolded. The use of qualitative methods provided a rich, nuanced foundation for deriving transferable lessons and policy-relevant insights. To ensure participant confidentiality, all data collected from the hubs were anonymised. To provide greater contextual grounding for the empirical findings, additional information on the specific characteristics and activities of the participating bioeconomy hubs is included. ^58^ Rather than focusing solely on structural differences such as country size, the analysis is complemented by concrete examples of hub activities, including the types of biomass resources, value chains, and innovation practices addressed in each region. These include, for instance, activities related to agricultural residues, circular farming practices, biomass valorisation, and region-specific bio-based applications.

Table 1 presents selected examples of hub activities alongside key analytical dimensions, such as stakeholder engagement formats and perceived impacts. This allows abstract concepts to be interpreted in relation to specific bioeconomy practices and regional contexts. The table is not intended to provide an exhaustive mapping but rather to illustrate how general patterns observed across the hubs relate to concrete implementation experiences and sector-specific challenges.

The analytical categories used in Table 1 structure the interpretation of hub activities. “Linked principles” refer to the underlying mechanisms associated with implementation: co-creation denotes collaborative, multi-actor development of activities and value chains; learning by seeing captures demonstration-based formats such as study visits; innovation adoption describes shifts in practices and mindsets towards bio-based solutions; and perceived value refers to tangible economic, environmental, or social benefits motivating participation. “Evidence types” indicate the empirical basis of each example: directly observed (implemented activities), reported by participants (stakeholder perceptions), and descriptive/project-based (planned actions and expected impacts from project documentation).

**Table 1.** Hubs with key analytical dimension: stakeholders, engagement formats, perceived impacts.

Hub/Region	Bioeconomy Activity (Specific)	Resources/Technologies	Stakeholders	Observed/Expected Impact
**Bretagne** & **Pays de la Loire** Regional HUBs (FR)	B2B Matchmaking during Hub Kick-off meeting in Nantes. International study visit between SPRING and VEGEPOLYS VALLEY.	Food and non-food plant-based by-products. Hemp processing and plant production.	34 stakeholders including private sector (Agrial, Upcyclink), research (INRAE), and territorial collectivities. SPRING delegation, 6 European organisations from Hemp Club, and French cluster representatives.	Observed: 37 matchmaking sessions conducted to identify synergies and valorise co-products. Knowledge exchange and networking between Italian and French hub actors.
	Linked Principle: Co-creation/stakeholder collaboration; Learning by seeing Evidence Type: Directly observed			
**Irish Regional** HUB (IE)	Study visit to the National Bioeconomy Campus in Lisheen. Farm Walks (Planned activity).	Bioprocess pilot plant, dairy processing residues valorisation. Small-scale bio-based solutions on climate-neutral dairy farms.	Co-operatives, banking organisations, and dairy industry representatives led by IBF and Tipperary County Council. Farmers and bioeconomy researchers.	Observed: Stakeholders shown what can be done with feedstocks to valorise waste. Expected: Facilitated discussions on practical implementation of bio-based solutions on-site.
	Linked Principle: Learning by seeing Evidence Type: Directly observed and reported by participants			
**Marche** Regional HUB (IT)	Study visit to the agricultural consortium of Jesi.	Agricultural production and innovative processing equipment.	AMAP officials, agricultural enterprises (GO RICREA, GO BHS), and researchers.	Observed: Exchange of experiences and good practices between small-scale producers.
	Linked Principle: Learning by seeing Evidence Type: Directly observed by Hub coordinator			
**BIOEAST HUB** CZ Charles Spa (CZ)	Revitalisation of Pond Medard as a strategic project for landscape repurposing.	Post-extraction landscape, biogas processing technology.	Industrial player Sokolská uhelná, a.s., and regional administration.	Expected: Diversification of revenue away from coal, job creation for younger generations, and carbon footprint reduction.
	Linked Principle: Innovation adoption/mindset change Evidence Type: Reported by participants			
**Puglia** Regional HUB (IT)	Development of specific supply chain working groups and establishment of a Regional Technical Table on the Bioeconomy. Study visit to Pedone Factory.	Agroenergy, biofertilizers, bioactive compounds, and hemp processing. Circular bio-economy production facilities.	Public Administrations (Department of Agriculture), companies, trade associations, and consultants.	Expected: Sustainable development in the region, new employment opportunities, and creation of a permanent legacy for decision-making. Promoting development by showing concrete real-world examples.
	Linked Principle: Co-creation/stakeholder collaboration; Learning by seeing Evidence Type: Descriptive/project-based			
**Lombardy** Regional HUB (IT)	Implementation of industrial symbiosis partnerships for short supply chains.	Agri-food waste, rice and tomato residues, corn stalks.	Farmers, processing industry (e.g., Mogu srl), and researchers (CNR, CREA).	Expected: Elimination of waste, reduction of transport impact, and creation of high value-added products.
	Linked Principle: Perceived value/tangible benefits Evidence Type: Descriptive/project-based			
**Latvia** Regional HUB (LV)	Value chain development for waste wood ash and biochar usage.	Wood ash from boiler houses, low quality peat, biochar residuals, pelleting technology.	Private forest owners, boiler house operators, and agricultural holdings.	Expected: Production of local bio-fertilizers for individual tree fertilization in plantations.
	Linked Principle: Perceived value/tangible benefits Evidence Type: Descriptive/project-based			
**Centru** Regional HUB (RO)	Implementation of small-scale biomass district heating systems (DHS).	Wood industrial residues, agrobiomass, modern boiler technologies.	Local authorities (LAGs), timber producers, and households.	Expected: Reduction of heating costs for public buildings and significant savings in local budgets.
	Linked Principle: Perceived value/tangible benefits Evidence Type: Descriptive/project-based			

**Figure 1:**
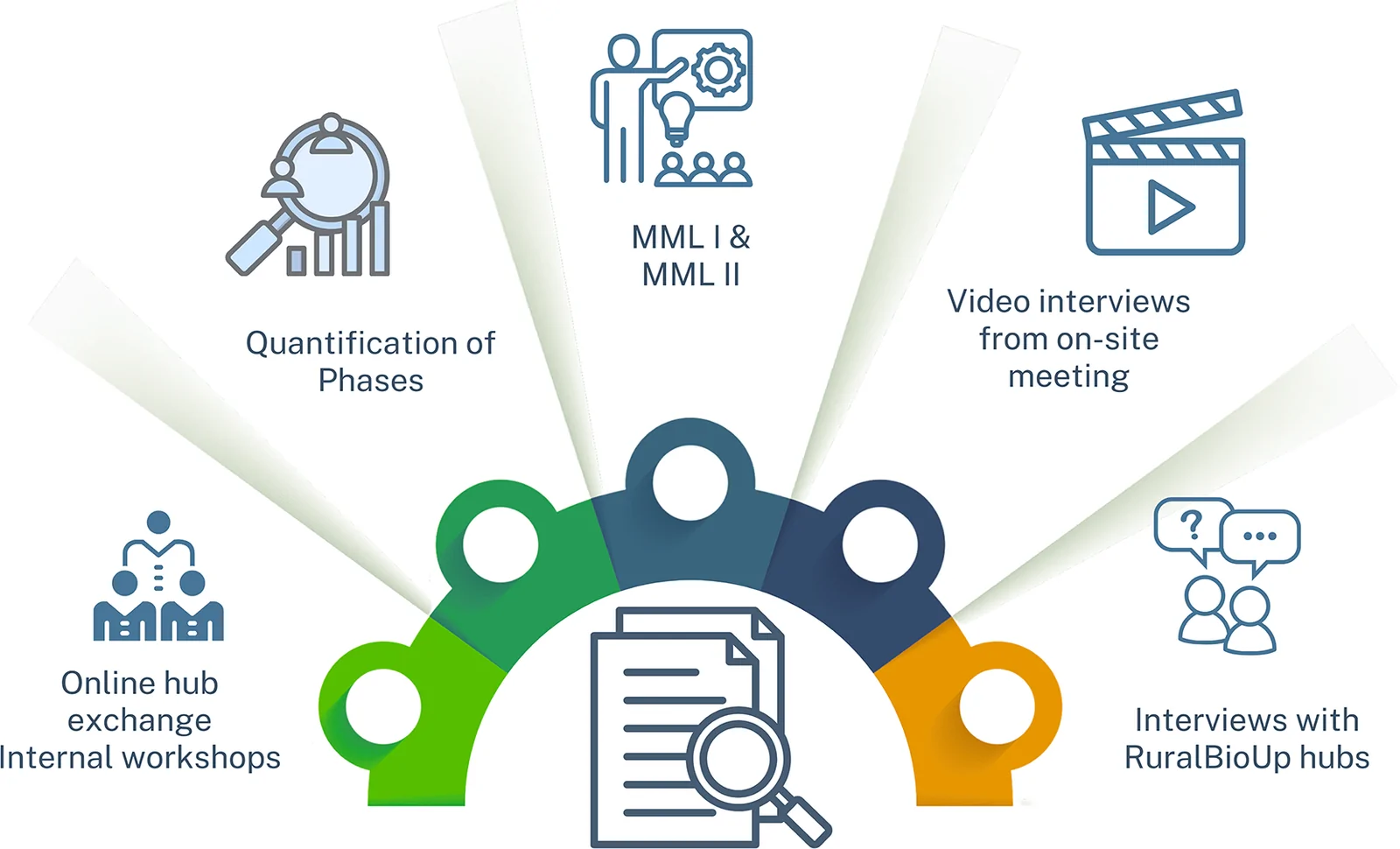
Overview sources for qualitative analysis.

To extract findings from the implementation of the RuralBioUp hubs, we applied a comprehensive, multi-layered analysis covering the entire project duration from its launch in October 2022 through to June 2025. The qualitative and quanitative data collection generated a large number of sources Figure 1 guiding the entire process of planning, implementing, and evaluating the bioeconomy hubs. The diversity and volume of inputs - ranging from online hub exchange meetings, two mutual learning (MML) workshops, one interactive ranking via Mentimeter to seven semi-structured interviews with hub facilitators, enabled a well-rounded and in-depth understanding of hub dynamics.

This multi-method approach enabled the capture of both structured feedback (via interviews, exchange meetings, and workshops) and more spontaneous insights (via ranking via Mentimeter sessions). The combination of these data sources provided a well-rounded view of the project’s achievements, challenges, and areas for improvement.

The collected qualitative data sets of the interviews were analysed using MAXQDA, a qualitative data analysis software widely used in social sciences. MAXQDA enabled us to systematically code and categorise qualitative inputs to identify recurring themes, patterns, and insights. In qualitative research, coding refers to the process of organising and labelling segments of textual data based on emerging themes or concepts. These codes help researchers interpret the data systematically and uncover underlying structures or meanings. The software’s advanced features supported for the integration of multiple coding lists. For the analysis, we started with nine overall deductive codes (pre-defined based on theory or research questions) derived from the online hub exchange meetings. These overall codes were further classified in total of 36 inductive codes (developed for themes that emerged during the coding process) according to relevant dimensions, offering a nuanced understanding of aspects that influenced the implementation, governance, socio-economic functionalities, issues and facilitating aspects of the RuralBioUp hubs. This process was intended to ensure consistency of defined key topics while also allowing flexibility for new, emerging topic areas within a structured analytical framework.

Our analysis of the data sets resulted in a total of 240 coded segments, capturing a wide spectrum of insights gathered throughout the study. The research results derived from the qualitative analysis were further refined, detailed, and examined through two MML workshops. The MML workshops allowed for cross-regional comparison, clarification of ambiguous findings, and identification of additional contextual factors that had not surfaced during individual interviews. They also contributed to the triangulation of results and supported the credibility and relevance of the emerging categories by allowing participants to confirm, nuance, or challenge early interpretations.

## Results and findings

The empirical findings in response to the three research questions, focusing on: (RQ1) hub facilitators perceptions of effective and ineffective actions in establishing bioeconomy hubs; (RQ2) the systemic conditions that facilitate or hinder the practical implementation of co-creation; and (RQ3) strategies for fostering innovation and initiating mindset shifts in traditionally structured rural environments indicated several aspects that are outlined in the following section:

### Hub facilitators perceptions of effective and ineffective actions for establishing regional bioeconomy hubs (RQ1)

Based on analised data the findings highlight the importance of engagement and sustained participation of local actors and suggest that this is associated with factors beyond well-intended policy design or funding. Understanding which actions are perceived as effective or ineffective is important for interpreting how strategic intentions translate into operational outcomes. Accordingly, strategies perceived as effective tended to centre on practical, tangible benefits and direct, personal engagement, such as study visits and targeted, co-organized events. In contrast, actions perceived as too generic, administratively burden some or lacking clear, immediate value, were less effective in supporting stakeholder participation and commitment.

***The importance of perceived value of participation by stakeholders***

According to the hub facilitators, stakeholder engagement appears to be influenced not only by the theoretical relevance or strategic ambition of an action, but also by its perceived practical value. Local actors (entrepreneurs, farmers, SME representatives, or municipal officers) evaluate initiatives based on immediate relevance, tangible benefits, and the degree to which they feel directly addressed. This lived experience perspective helps to explain observed differences in levels of commitment across hubs. Actions perceived as effective were typically described as hands-on, directly useful, and personally meaningful, while those perceived as ineffective were described as abstract, burdensome, or misaligned with local needs and capacities.

***Effective actions: What worked according to hub facilitators***

**Practical and tangible activities**

A consistent theme in hub facilitators’ feedback was the importance of concrete, experiential learning formats, which were perceived as more engaging than abstract presentations or theoretical sessions.

Study visits (visits of hub members to another bioeconomy hub or facility) emerged as a particularly prominent engagement tool. Participants described them as valuable opportunities: “ *This is one of the best ways to include and share information and to be practical.*” (H7). This suggests that direct exposure to real-life practices are associated with higher perceived effectiveness. These visits were described as helping to demystify bioeconomy concepts and support learning by illustrating what is achievable in practice. Participants emphasized that such visits were perceived as useful because they provided concrete insight into development trajectories (H4).

Business matchmaking events that connected biomass producers with potential users of their by-products were also reported as effective. These formats were perceived as immediately useful, as they were associated with generating tangible economic opportunities and fostering new collaborations.

Events that fostered visibility and recognition also were reported to play a motivational role. Stakeholders responded positively to being featured in promotional materials or invited to speak at events. Such exposure was perceived as both recognition and an opportunity to build professional reputation within the regional bioeconomy context.

**Targeted and collaborative events**

The format and context in which events were organised were reported to influence participation and perceived impact:

Co-organising events with other initiatives were described as enhancing legitimacy and visibility: “ *Co-organizing worked well. It attracted more participants and allowed for broader engagement”* (H1). Facilitators noted that such formats were perceived as more embedded in community structures rather than as isolated interventions. Participation was reported to be higher when events were integrated into existing networks.

On-site training sessions on specific, pre-identified were described as particularly effective when delivered in local languages. The perceived effectiveness was linked to contextual relevance and accessibility.

Direct, personal outreach methods were reported as highly effective, particularly in more conservative or traditionally structured areas. *“A ‘door-to-door’ engagement approach was effective in the hub’s country, directly visiting businesses instead of relying on emails that might get lost in spam”* (H7). This approach was associated with facilitating the building of personal trust and the reduction of perceived distance between project teams and stakeholders.

**Strategic networking and support mechanisms**

Effective engagement appeared to be linked to leveraging existing structures and addressing stakeholder needs.

Utilising established networks was described as important for building legitimacy and outreach. These intermediaries were reported to function as bridges between innovation initiatives and local communities: *“Success hinges on the collaboration of local authorities, academia, industry, and civil society, driven by innovation”* (H2).

Access to information on funding opportunities was frequently cited as key motivating factor. Stakeholders showed interest in how participation could lead to financial benefits, which, in turn was linked to higher engagement.

A key theme that emerged from the findings was the need for economic sustainability in the bioeconomy hubs. Hubs that demonstrated clear economic incentives for participation, such as access to funding or market opportunities, were more likely to experience long-term stakeholder engagement. Financial stability allows hubs to invest in long-term goals, such as expanding supply chains, strengthening partnerships, and scaling up innovations.

***Ineffective actions: What didn’t work - and why***

While several strategies supported engagement, facilitators also identified actions that were perceived as less effective or counterproductive. Four recurring patterns were identified: lack of contextual relevance, administrative burden, misalignment with local communication norms, and overextension across multiple value chains.

Generic online trainings delivered in English were frequently reported as ineffective: *“… generic workshops in English didn’t attract participants …”* (H1). These sessions were described as too broad or disconnected from local needs, and in some cases language barriers further limited participation.

Activities perceived as administratively burdensome (e.g. questionnaires) were reported to reduce engagement, particularly when benefits were unclear.

Top-down communication approaches were described as less effective in contexts where trust in external institutions is limited. In such cases, the identity of the messenger was reported to influence reception as much as the message itself.

Overextension across multiple value chains was identified as a limiting factor. When initiatives addressed too many topics simultaneously, outcomes were described as becoming less tangible: *“… having three value chains is quite a lot …”* (H7). A narrower focus was reported as being associated with clearer outcomes and stronger engagement.

In summary, the findings suggest that local actors tend to respond more positively to actions that provide clear, concrete benefits, practical learning opportunities, and direct personal interaction, particularly when focused on a limited number of value chains. Strategies such as study visits and B2B (business to business) matchmaking were reported as effective in supporting engagement, while generic online activities and administrative complexity were associated with lower participation. Overall, approaches that are tailored to local contexts, leverage existing networks, and demonstrate tangible benefits were more frequently perceived as effective by participants.

### Systemic conditions facilitating and hindering the translation of co-creation into practice in regional bioeconomy hubs (RQ2)

The successful implementation of co-creation in regional bioeconomy hubs is associated not only with the design of individual activities but also with a broader set of systemic conditions. These enabling factors operate at institutional, relational, and contextual levels and provide conditions under which specific engagement strategies can be meaningfully executed. Across regions participating in the RuralBioUp project, several common enablers were identified as influencing the emergence of conditions in which co-creation could take place.

***Embeddedness in local contexts and bottom-up framing***

A fundamental enabler was described as the alignment of co-creation efforts with local priorities, mentalities, and production realities. Co-creation processes were reported to be more effective when grounded in an understanding of local culture, values, and economic structures. A bottom-up approach was associated with enabling stakeholders to recognise their own challenges, interests, and knowledge in the agenda-setting process, rather than perceiving it as externally imposed. This contextual sensitivity is widely discussed in literature as an important condition for authentic collaboration.

**Trust-based relationships and network capital**

The strength of existing local networks and trust-based relationships was reported to influence the feasibility of co-creation. Hubs that could build on pre-established platforms, such as farmer associations, local innovation intermediaries, or municipal networks, were reported as more successful in securing engagement. These networks functioned as social infrastructure for knowledge exchange and were associated with reducing the perceived risk of participation. In several regions, ‘hub contact points’ (trusted individuals or organisations embedded in the community) were reported to be instrumental in enabling dialogue and reducing scepticism: *“A key strategy is maintaining good working relationships. If people trust you and your work, they’ll participate. The most challenging part is targeting people outside your existing network and keeping them engaged”* (H7).

**Local facilitation and proximity of coordination**

A dedicated local facilitator was identified as important factor. Unlike external experts, local facilitators were described acting as boundary spanners, translating between policy language and everyday concerns. Their proximity was associated with enabling informal, flexible, and responsive engagement, which in turn was linked to greater credibility and continuity in stakeholder relationships.

**Governance flexibility and institutional support**

Co-creation is inherently iterative and adaptive. Accordingly, implementation was reported to be influenced by institutional flexibility – that is, the ability of projects and governing structures to adapt to stakeholder input, adjust timelines, and recalibrate strategies as needs evolve. Rigid governance mechanisms, in contrast, were described as constraining co-creation processes. Supportive institutional environments were associated with allowing co-creation processes to remain, participatory, and responsive to changing circumstances.

**Visibility of value and incentive alignment**

While co-creation is based on collective processes, individual stakeholders were reported to consider personal or professional value when deciding whether to participate: “ *They needed to see a ‘real impact or real change’ and know ‘what value you’re bringing to their lives*” (H7). Hubs that clearly communicated potential benefits, such as access to funding, recognition, or new market opportunities, were associated with stronger engagement. When stakeholders perceived that co-creation contributed to their own goals, they were more likely to invest time and effort in collaborative processes. H6 further noted that *“… concrete opportunities matter more than just networking …”* (H6), and that stakeholders often sought funding opportunities (H6, H3).

***Structural barriers hindering co-creation in practice***

While co-creation holds strong conceptual appeal and is widely promoted in policy frameworks, its implementation in rural bioeconomy contexts has been reported to be hindered by systemic constraints. These barriers are not simply logistical challenges, but they have been described as reflecting deeper structural issues, including institutional inertia, relational deficits, and misaligned incentives. Insights from the RuralBioUp project illustrate how these barriers manifest in practice and how they may undermine the co-creative potential of regional bioeconomy hubs.

**Time scarcity and capacity constraints**

One of the most consistent obstacles to co-creation was reported to be the limited availability and capacity of stakeholders, particularly those from the private sector such as farmers and small business owners: *“It is very hard to involve the private sector, especially farmers* (H7). Many were described as operating with limited surplus time or resources and therefore were less able to engage in ongoing, iterative processes without clear and immediate benefit. This scarcity of time was reported to restrict both the breadth and depth of stakeholder involvement and may contribute to co-creation becoming episodic rather than sustained. Some facilitators also indicated that engagement was shaped by stakeholders’ economic capacity and ability to invest time, suggesting implicit feasibility constraints.

**Stakeholder scepticism and cultural resistance to change**

In several regions, co-creation efforts were reported to be affected by cultural conservatism and scepticism toward externally initiated innovation. Stakeholders, particularly in more traditional rural areas, were described as cautious about engaging with new initiatives unless they could identify concrete, long-term value for their own enterprises or communities. In some cases, scepticism was linked to past experiences with short-lived or top-down projects. Without deliberate trust-building and visible impact, co-creation may be perceived as a transient initiative disconnected from local concerns.

Equally important to the success of the hubs was their robustness in dealing with setbacks. Hubs that built in adaptive strategies, such as safe experimentation and peer learning networks, were better equipped to recover from failures and remain functional. For instance, hubs embraced a trial-and-error approach to testing bio-based solutions, allowing for controlled experimentation without significant financial risk to participants. This approach not only mitigated the impact of failures but also provided valuable learning experiences that enhanced the hub’s resilience and capacity for future innovation.

**Ambiguity around purpose and value proposition**

Hub facilitators reported that a lack of clarity regarding the mission, scope, and added value of the bioeconomy hub was perceived as a barrier to engagement. Stakeholders often reported difficulty in distinguishing the hub from existing clusters or support platforms, particularly when communication was vague or overly technical. This conceptual ambiguity was associated with reducing perceived relevance and contributing to disengagement. Effective co-creation has been described in the literature as benefiting from a clearly communicated and shared understanding of purpose, established early in the process.

**Geographical and infrastructural limitations**

In large or sparsely populated regions, geographical distance, and limited transport infrastructure were reported to make in-person collaboration more difficult to sustain. While digital tools were employed to bridge these gaps, online engagement formats, particularly when conducted in English or lacking interactivity, were often perceived as less effective in attracting meaningful participation. The digital divide, language barriers, and format fatigue were described as contributing to a sense of disconnection from the process and limiting opportunities for trust-based relationship building.

**Institutional rigidity and power asymmetries**

Co-creation is often described as being facilitated by flexible and inclusive governance environments. However, some hubs were reported to face institutional rigidity that limited the responsiveness of project structures. Predefined deliverables, strict timelines, and procedural constraints were described as reducing the possibility for adapting to stakeholder input or shifting priorities. Additionally, power asymmetries between stakeholder groups were reported to result in imbalances in decision-making, where co-creation was perceived as symbolic rather than substantive. When stakeholders perceived that decisions were made elsewhere, engagement was reported to decline and perceived legitimacy to be reduced.

### Enabling bioeconomy adoption in traditional rural areas (RQ3)

Introducing bioeconomy and innovation in traditionally conservative rural regions has been described as involving cultural and psychological change. Resistance has been reported to stem from deep-rooted worldviews and past experiences rather than disinterest; accordingly, top-down approaches have often been described as less effective. Instead, strategies that are associated with building trust, relevance, and co-adaptation, integrating new ideas into familiar contexts, were reported as more effective in the studied cases. Co-adaptation refers to a process in which new ideas are introduced in ways that are sensitive to local norms, while communities gradually adjust their practices.

Drawing on insights from the RuralBioUp project, mindset change was described as requiring patience, humility, and sustained engagement.

***Key Strategies for enabling innovation through co-adaptation***

Building trust and awareness was identified as an important strategy in conservative rural contexts. Approaches aimed at making new ideas more relatable and contextually grounded were reported as facilitating implementation:

**Co-creation grounded in local realities**

Rather than introducing fully formed solutions, initiatives perceived as successful engaged stakeholders in co-creating innovations adapted to local strengths, opportunities, and challenges. H5 emphasised the importance of *“… understanding the local context …”* (H5). This co-creative process was associated with improving solution fit and with contributing to legitimacy while reducing resistance among stakeholders.

As noted in previous findings, co-creation in conservative regions was reported to be more effective when starting from existing practices, community structures, or resource flows that align with bioeconomy goals. This approach was associated with fostering continuity, trust, and commitment by embedding innovation within familiar environments rather than replacing them. *“Younger stakeholders might be more receptive …”* (H7).

Across the findings, a co-adaptive approache were consistently described as important. This approach was reported as particularly relevant in traditional settings. By embedding change within familiar reference points and allowing time for iterative learning, co-adaptive approaches were associated with supporting bioeconomy transitions.

**Practical demonstrations and Peer-to-Peer learning**

Practical, visible examples, such as study visits, were frequently described as effective in supporting mindset change. These demonstrations were reported to enable stakeholders to observe innovations in action. Peer-to-peer learning formats, such as “farmers training farmers,” were identified as particularly influential, as they rely on trusted local actors and were associated with reducing perceived risks while reinforcing legitimacy. Stakeholders were reported to be more likely to consider adopting new practices when observing their implementation in comparable contexts.

**Connecting innovation to familiar practices**

Innovation adoption was reported to be more likely when framed in relation to existing practices rather than presented as entirely novel. H2 noted the importance of aligning initiatives with local priorities and avoiding top-down imposition (H2). For instance, composting can be interpreted as an extension of traditional organic farming or waste reuse practices. This framing was associated with making innovation appear less disruptive and more evolutionary. According to hub facilitators, this approach was also described as helping to reduce the perceived unfamiliarity of bioeconomy concepts. By localising terminology and linking new practices to culturally resonant ideas, bioeconomy hubs were reported to lower entry barriers and to support a sense of familiarity and ownership.

***Strategic engagement and incentives***

Strategic engagement and tailored incentives were identified as important factors associated with motivating participation in conservative rural contexts. Approaches that enable early engagement and demonstrate tangible benefits were reported as facilitating implementation.

**Early engagement of key stakeholders**

H3 observed that direct, personal, face-to-face engagement was perceived as more effective for building trust and attracting participation, as emails and online methods were reported as less effective among *“old fashioned thinking people”* (H3) in their region. Another element identified as important is the strategic involvement of key stakeholders, such as respected local figures or multipliers, (e.g. farmers’ association leaders, teachers, or regional development representatives). Their endorsement was reported to support the acceptance of new ideas, particularly in regions where hierarchical or communal authority plays a role. Including younger generations - as both users and communicators of innovation, was also described as potentially acting as a social bridge between traditional mindsets and forward-looking practices.

**Demonstrating tangible, local benefits**

Successful strategies were reported to emphasise that the benefits of innovation are visible, relatable, and locally adapted. Stakeholders were described as more likely to engage with new practices when they could observe positive outcomes, such as increased income, reduced waste, or improved crop resilience, within their own community. Innovation that delivers concrete, short-term value, even in modest ways, was associated with supporting the confidence in longer-term transformation processes.

***Strategies overcoming barriers to mindset change***

Even with the right strategies, the process of shifting mindsets in traditionally structured rural environments has been reported to involve persistent and deeply rooted challenges, including risk aversion, economic uncertainty, and emotional attachment to established practices. Addressing these barriers has been described as involving not only engagement strategies but also long-term commitment, cultural sensitivity, and the use of information to build trust and confidence.

**Patience and vision**

According to the hub facilitators, one important factor in overcoming resistance was identified as recognising that mindset change is a gradual process: *“… they did not expect a large immediate effect from the RuralBioUp project, understanding that results would develop over time”* (H2). In conservative regions, stakeholders, particularly small-scale farmers and entrepreneurs, were described as often hesitant to experiment with new business models or value chains unless they perceived clear returns. This caution can be interpreted as a rational response to perceived risks and limited margins for failure.

Accordingly, impatience or pressure for quick results was reported as potentially counterproductive. Instead, projects were described as benefiting from cultivating a shared vision for change and maintaining consistent communication over time, even when immediate engagement remained limited. This includes listening to local concerns, setting realistic expectations, and maintaining a visible presence in the region. Trust was described as developing incrementally, with more transformative discussions emerging over time.

**Data-driven decision making**

Another approach reported as effective in addressing scepticism involved the use of data and evidence-based planning. When potential innovations, such as new value chains or biomass uses, were supported by regional studies, economic feasibility analyses, and sectoral data, stakeholders were more likely to consider them legitimate and viable.

Presenting data in an accessible and context-specific way, such as through tools like RuralSpot, was described as helping to ground discussions in locally relevant evidence. For example, desk studies that highlight available regional feedstocks or map supply-demand gaps were reported to support stakeholders in identifying opportunities. This was associated with reducing perceived risk and supporting the credibility of proposed changes.

**A strategic, culturally sensitive approach**

Overcoming barriers to innovation in conservative rural environments has been described as involving processes that extend beyond persuasion alone, including supporting communities in navigating change. H2 observed that their region, influenced by its history, led people to be *“rather shy and conservative”* (H2) when engaging with new business ventures.

This was described as requiring strategies that balance empathy with leadership and long-term perspectives with short-term outcomes. Approaches combining patience, relevance, and transparency were associated with shifting stakeholder perspectives over time. Through practical demonstration, peer-led engagement, and locally grounded co-creation, stakeholders were reported to increasingly perceive innovation not as an external imposition but as a potential opportunity to shape regional development.

## Discussion

The findings should be interpreted in light of the study’s analytical focus on co-creation and stakeholder engagement, which are examined here as context-dependent factors associated with implementation outcomes rather than as universally optimal approaches. Therefor this study seeks to contribute to the literature on regional bioeconomy hubs by extending evidence from earlier initiatives such as BIOVoices ^1^ and BioBridges. ^2^ While those projects largely emphasized knowledge sharing and policy dialogue, RuralBioUp provides empirical examples of co-creation strategies that are associated with stakeholder ownership, including conservative rural contexts. By combining empirical evidence from nine regional hubs with a focus on co-adaptive approaches, this work addresses an identified gap in the bioeconomy hub literature.

In doing so, it highlights the interplay between stakeholder perceptions of impactful actions, co-creation practices, and mindset shifts in shaping the establishment and operation of regional bioeconomy hubs. The findings indicate that social and institutional dynamics influence the translation of bioeconomy concepts into locally embedded innovations. This contributes to academic discussion on how context-sensitive, trust-based interventions may support rural sustainability transitions.

### Linking perceived effectiveness to implementation outcomes

Our results suggest an interplay between the perceived effectiveness of actions and implementation outcomes. Consistent with innovation systems and transition theory, ^53^ stakeholder engagement appears to be influenced not only by the inherent quality or innovativeness of an intervention but also by its perceived relevance, legitimacy, and sense of ownershiBroader inclusiveness may therefore emerge progressively over time, following initial engagement by more motivated or resourceful stakeholder groups.

While inclusiveness and co-creation are widely associated with legitimacy, stakeholder ownership, and social sustainability, they do not inherently ensure the economic viability of hub activities. Empirical and theoretical work on bioeconomy transitions emphasises that sustainable innovation requires the alignment of environmental, social, and economic dimensions, which may at times involve trade-offs rather than synergies. In practice, regional hubs operate under constraints of limited time, resources, and market demand, which can restrict the feasibility of engaging all stakeholder groups equally across all phases of the innovation process.

In this sense, economic sustainability is not an alternative to social sustainability but a necessary condition for its long-term realisation. Participation strategies may therefore need to prioritise stakeholders who are both willing and able to contribute to viable value chains, while broader inclusiveness can emerge more gradually over time. Governance arrangements such as cooperative ownership models, local partnerships, and other mechanisms that distribute costs, benefits, and risks more evenly have been described as potential ways to reconcile these dimensions. Such arrangements may strengthen local commitment while simultaneously supporting business viability and long-term resilience of bioeconomy initiatives.

From the perspective of diffusion of innovation theory, ^53^ engagement in bioeconomy transitions is likely to occur unevenly across stakeholder groups. Early adopters and “coalitions of the willing” may play a disproportionate role in initiating change, while broader participation may emerge gradually over time.

Actions that resonate with local economic and social realities, are co-endorsed by trusted local actors, and allow stakeholders to see themselves reflected in the process (e. g. through co-design or public recognition) were associated with deeper commitment and sustained involvement. This aligns with findings in participatory governance literature emphasizing that legitimacy and local ownership are considered important for sustaining innovation uptake. ^27^

Conversely, activities perceived as generic, administratively complex, or disconnected from immediate local interests were reported to be associated with scepticism and disengagement and may affect trust in bioeconomy initiatives. This supports previous research highlighting limitations of “one-size-fits-all” approaches and the relevance of tailoring strategies to local contexts. ^59^ In practical terms, these findings suggest that bioeconomy hubs may benefit from designing engagement formats that involve stakeholders as co-creators rather than passive recipients, with attention to tangible benefits such as learning opportunities, visibility, and access to funding. This is consistent with stakeholder-centric models such as co-creation in sustainability transitions.

### Institutional and relational preconditions for co-creation.

The study further elucidates the conditions associated with translating co-creation theory into practice. Consistent with broader literature on innovation ecosystems, ^21^ ^,^ ^28^ co-creation is described as involving not only participatory methods but also enabling governance environments characterised by institutional flexibility, equity, and sustained engagement. The identified structural barriers, such as stakeholder time constraints, scepticism, and logistical challenges, indicate the presence of institutional inertia and trust deficits that may constrain collaborative processes.

These findings suggest the relevance of relational trust-building and transparent, stakeholder-centred communication in co-creation processes. Governance arrangements may benefit from allowing adaptive management, including responsiveness to local power imbalances and socio-cultural specificities. Such contextual sensitivity is widely discussed as important for fostering innovation environments that are perceived as legitimate and accessible to relevant stakeholder groups. In this respect, the results provide empirical support for arguments in transition studies that emphasise the need to connect institutional frameworks with grassroots dynamics. ^18^

### Mindset shifts as a foundation for rural bioeconomy adoption

Finally, the research indicates that mindset change in conservative rural environments is often described as a gradual and iterative process shaped by trust, dialogue, and co-learning rather than rapid technological shifts or external interventions. This resonates with transition literature emphasising the socio-cultural dimensions of systemic change. ^18^ Bioeconomy adoption was reported to be more likely when innovations were framed as extensions or adaptations of existing local practices, supported by peer learning and visible local benefits. Beyond social acceptance and perceived relevance, the findings also point to the importance of robustness in innovation processes. In risk-averse rural environments, stakeholders are not only concerned with the potential benefits of new practices but also with the consequences of failure. ^55^ The willingness to experiment with bioeconomy innovations appears to be closely linked to the existence of mechanisms that reduce individual exposure to risk. ^20^ ^,^ ^55^ Such mechanisms may include pilot and demonstration projects, cooperative or collective implementation models, phased adoption strategies, and access to public support schemes that buffer potential losses. These arrangements allow stakeholders to test innovations under controlled or shared-risk conditions, thereby lowering the threshold for initial engagement. ^20^ In this context, co-creation processes and bioeconomy hubs can be understood not only as platforms for knowledge exchange but also as enabling environments for “safe experimentation”. ^20^ ^,^ ^30^ By distributing risks across multiple actors, facilitating peer learning, and embedding innovation within trusted networks, they may contribute to increasing stakeholders’ confidence to engage with new practices, even under conditions of uncertainty. ^42^ ^,^ ^46^

The emphasis on co-adaptive approaches over disruptive change was observed in the studied contexts, suggesting the relevance of embedding innovation within familiar identities and narratives. However, this does not imply that disruptive innovation is inherently undesirable. Rather, the findings indicate that in the analysed rural contexts, incremental and co-adaptive approaches were more readily accepted by stakeholders. In other contexts, particularly where institutional inertia is lower, or market incentives are stronger, more disruptive innovation pathways may also be feasible and desirable. This was associated with facilitating acceptance by aligning with community values and reducing perceived risks. Such processes were described as requiring time and sustained engagement, as economic incentives alone may not be sufficient to drive transformation in contexts where social cohesion and tradition play a significant role.

The findings suggest that regional bioeconomy hubs can be potentially act as intermediaries that connect existing practices with emerging innovation pathways, supporting development trajectories that are rooted in local contexts while engaging with broader sustainability transitions. ^60^

### Implications for policy and practice

The findings of this study suggest that implementing bioeconomy hubs in rural regions involves not only technical or administrative aspects, but also socio-institutional processes related to trust, local relevance, and community ownershiFor both practitioners and policymakers, this has implications for how rural bioeconomy transitions may be designed, supported, and evaluated.

First, engagement was reported to be more effective when rooted in practical, context-specific formats that demonstrate visible value to participants. Actions such as study visits, peer-led demonstrations, and co-organised events in local languages were consistently perceived as more effective than generic trainings or top-down communications. These formats were associated with higher levels of perceived inclusion and ownership by stakeholders, as they connected innovation to familiar experiences and everyday concerns. In contrast, excessive administrative demands, unclear messaging, and attempts to address multiple value chains were reported to reduce perceived effectiveness and participation.

Secondly, trust-building and local facilitation were identified as important relational factors for adapting activities to local needs and enabling inclusive engagement processes. Local facilitators were described as playing a key role in mediating between stakeholders, supporting trust-building, and enabling context-sensitive interaction.

Third, flexible and adaptive governance arrangements were identified as equally important at the institutional level, particularly for enabling iterative feedback processes, adjusting activities over time, and accommodating context-specific needs.

Also, innovation in traditionally conservative rural settings was reported to be more readily adopted when approached through culturally sensitive, co-adaptive strategies. Rather than promoting disruptive change, strategies that built on existing knowledge, values, and relationships were perceived as more effective. Framing bio-based innovations as extensions of traditional practices, involving respected community members early, and demonstrating visible, place-based benefits were associated with fostering legitimacy and reducing resistance. Stakeholders were reported to respond more positively when participation aligned with their professional goals, offered recognition, or enabled access to opportunities.

Finally, fostering long-term mindset change was described as requiring sustained engagement over time. Transformative processes in rural bioeconomy contexts were characterised as cumulative and relational rather than linear. Accordingly, policies may benefit from moving beyond one-size-fits-all models and instead supporting adaptive implementation pathways that allow for local experimentation, trust-building, and shared narratives of progress. Thus, the support of “safe experimentation” environments by funding pilot projects, facilitating cooperative risk-sharing models, and providing financial or institutional buffers allow stakeholders to test innovations without bearing full individual risk.

**Figure 2:**
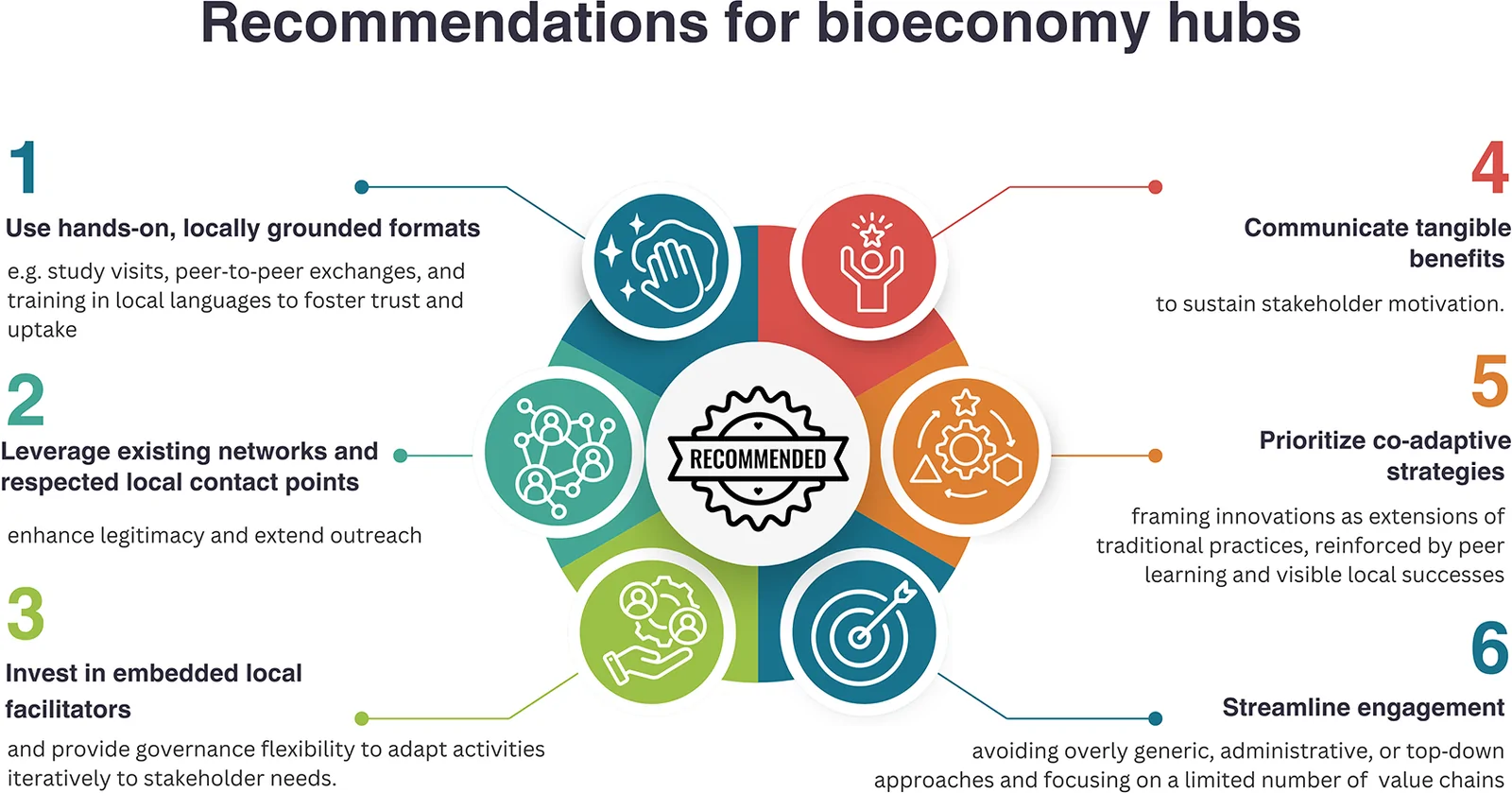
Overview recommendations hub establishment.

Consequently, policy recommendations from a practical perspective Figure 2 tackles:

**Use hands on, locally grounded formats** such as study visits, peer to peer exchanges, and training in local languages to foster trust and uptake.

**Leverage existing networks and respected local contact points** to enhance legitimacy and extend outreach, especially in conservative rural settings.

**Invest in embedded local facilitators** and provide governance flexibility to adapt activities iteratively to stakeholder needs.

**Communicate tangible benefits** (e.g. funding access, recognition, business opportunities) to sustain stakeholder motivation.

**Prioritise co adaptive strategies** that frame innovations as extensions of traditional practices, reinforced by peer learning and visible local successes.

**Streamline engagement** by avoiding overly generic, administrative, or top-down approaches and focusing on a limited number of high potential value chains.

By aligning bioeconomy initiatives with the lived realities of rural communities and fostering trust-based, co-adaptive engagement, policymakers can support bioeconomy hubs as vehicles for context-sensitive and socially embedded forms of sustainable change. The findings of this study therefore may therefore provide concrete entry points for EU, national, and regional strategies to translate the bioeconomy from vision into practice.

The results extend beyond the operationalization of individual hubs, offering direct implications for EU and national bioeconomy policy design. They suggest the relevance of embedding co-creation and participation principles into policy frameworks, ensuring that strategies are not only top-down but also responsive to diverse local contexts. By demonstrating how trust-building, peer-to-peer learning, and context-sensitive adaptation are associated with higher levels of engagement in conservative rural regions, the evidence supports consideration of policies that foster inclusiveness, legitimacy, and long-term sustainability.

At the EU level, the findings are consistent with the objectives of the European Green Deal and the European Bioeconomy Strategy. Place-based and trust-driven approaches were reported as more effective in the studied contexts, suggesting that policies may benefit from promoting flexible funding instruments that allow local adaptation and prioritise multi-stakeholder collaboration. Incorporating these insights into forthcoming revisions of the EU Bioeconomy Strategy ^60^ and the Common Agricultural Policy (CAP) may strengthen alignment between high-level sustainability targets and local implementation.

At the national level, the evidence indicates the relevance of institutionalising local facilitation roles and peer-to-peer learning mechanisms within bioeconomy strategies. Supporting the training and funding of embedded facilitators, incentivising cross-sectoral networks, and reducing administrative complexity were identified as factors associated with increased participation and perceived legitimacy. National strategies may benefit from avoiding uniform, top-down models and instead supporting regional experimentation and co-adaptive processes that build on local capacities and traditions.

In addition, bioeconomy hubs designed with a clear focus on economic sustainability, integrating financial mechanisms such as market-driven models, cooperative ownership structures, or public-private partnerships are more likely to ensure long-term viability. To foster robustness and ensure long-term success, bioeconomy hubs should create a culture of safe experimentation. This includes providing peer-to-peer learning opportunities and adopting shared-risk models that allow stakeholders to experiment without the fear of failure. Hubs that embraced this approach were better equipped to recover from setbacks and adapt to changing conditions. This approach supports the resilience of the hub and its stakeholders over time. These approaches will not only enhance the economic feasibility of hubs but also help them withstand challenges and thrive in diverse local contexts.

Finally, the RuralBioUp findings indicate the need for further research, including longitudinal evaluation beyond 2025 to assess the enduring impacts of co-creation-based hubs, as well as comparative analyses in non-EU and Global South contexts to test their transferability under different socio-economic and institutional conditions. While this study focuses on EU-based implementation, reciprocity-based bioeconomy standards may create adjustment pressures for non-EU producers, particularly in developing countries where compliance costs, certification requirements, and institutional capacities differ significantly. In this context, EU sustainability-oriented standards can function as non-tariff barriers to trade. ^61^ This raises important questions regarding the inclusiveness and global equity of bioeconomy transitions, particularly in relation to access to international markets and the distribution of sustainability-related burdens.

Together, these considerations suggest that bioeconomy hubs can be understood as potentially acting as instruments for sustainable, inclusive, and climate-neutral rural development across Europe and beyond, provided that their broader international implications are taken into account. A related issue concerns how such standards are implemented in practice, particularly for non-EU producers. While international trade rules formally allow countries to meet regulatory objectives through different but equivalent approaches (e.g. SPS Agreement, Art. 4), ^62^ in practice, compliance with EU sustainability and bioeconomy standards often requires a high degree of alignment with EU rules. This may limit flexibility for third countries and create additional challenges for producers with different regulatory and institutional contexts. ^63^ ^,^ ^64^ At the same time, many sustainability standards focus on production methods that are not physically traceable in the final product (non-product-related production and process methods), which remain contested in WTO law as they challenge traditional interpretations of “like products”. ^65^ Together, these issues highlight the need to consider the broader international implications of EU bioeconomy policies when promoting inclusive and sustainable rural development.

### Limitations

While this study provides valuable insights into the development and implementation of regional bioeconomy hubs, several limitations should be acknowledged.

First, the research is based on a qualitative, multi-case design involving nine hubs across Europe. While this allows for in-depth, context-sensitive analysis, the findings are not intended to support statistical data. Instead, they offer analytical generalization and transferable lessons that may inform similar initiatives in comparable contexts.

Second, the study relies heavily on self-reported data from hub facilitators, collected through interviews, workshops, and participatory sessions. This reliance introduces the potential for bias, as facilitators may emphasize certain successes or underreport challenges. To mitigate this, findings were triangulated with multiple data sources - including Mentimeter feedback, and workshop results -and validated by experts in two MML workshops. While this enhances validity, interpretations remain subject to participant perception and selective memory.

Third, the project’s implementation timeframe (October 2022 – June 2025) may limit the ability to assess long-term impacts and sustainability of the hubs. The lessons learned reflect the hubs’ establishment and early operational phases rather than mature, fully embedded systems. Future research via mixed-methods research should therefore include longitudinal studies beyond 2025 as well as comparative analyses in non-EU rural contexts to assess long-term impacts and transferability.

Fourth, this study focuses on co-creation and inclusive engagement as observed in the RuralBioUp cases. It does not claim that these approaches are universally optimal. Alternative pathways, including more centralised or disruptive innovation strategies, may be effective in different institutional or market contexts. Further research is needed to compare these approaches across settings.

Lastly, the diversity of regional and cultural contexts, while a strength for capturing variation, also introduces complexity in comparison. Some findings may be shaped more by national policy frameworks, institutional structures, or funding environments than by the specific strategies applied within the hubs themselves.

Despite these limitations, the study offers empirically grounded, practice-oriented insights that contribute to the growing body of knowledge on participatory bioeconomy development in rural regions.

## Conclusions

This study contributes to a deeper understanding of how regional bioeconomy hubs can be effectively implemented in traditionally conservative rural contexts by illuminating the perceived successful actions for implementation, co-creation dynamics, and mindset shifts. It underscores that the success of bioeconomy initiatives hinges less on technological novelty and more on perceived relevance, legitimacy, and stakeholder ownershiActions that are practical, locally based, and visibly beneficial were shown to strengthen engagement, while top-down or generic interventions often led to disengagement or resistance.

Importantly, the research reaffirms the need for co-creation that is not merely a participatory method but a governance approach that requires institutional flexibility, trust-based relationships, and cultural sensitivity. Regional bioeconomy hubs function most effectively when they act as bridges, connecting innovation with local identities, aligning new ideas with existing practices, and fostering long-term, trustful relationships between actors.

For policy and practice, the findings suggest that future bioeconomy strategies should prioritize targeted, tangible engagement formats; utilize local languages and networks; and create visible incentives for participation. Policy frameworks should support governance models that enable local flexibility, reduce administrative burdens, and fund local facilitation to sustain grassroots engagement. Embedding innovation through co-adaptation rather than disruption emerges as a key strategy for overcoming scepticism and fostering meaningful participation.

With appropriate support structures, iterative learning processes, and policy alignment, regional bioeconomy hubs like those in RuralBioUp can evolve into resilient innovation ecosystems. These hubs not only catalyse local transitions but also hold the potential to contribute meaningfully to broader European goals, advancing the bioeconomy ways that are locally grounded, context-sensitive, and responsive to diverse stakeholder dynamics.

Still, future research should explore the long-term outcomes of such co-creative bioeconomy hubs, including their capacity to build local innovation ecosystems. In the same way, the exploration of the long-term structural change should become key topic for future research. Comparative studies across different governance settings and local differences could further clarify how institutional arrangements shape stakeholder engagement and innovation uptake. Additionally, more work is needed on the role and requested skills of intermediaries and facilitators in translating policy into practice, particularly in rural contexts where traditional values and risk aversion remain strong.

### Ethics and consent

All participants provided informed consent before taking part in the study. The process included a full briefing on the research purpose, use of contributions, data handling, and privacy protection. Participants were informed about the study objectives, the voluntary nature of their involvement, and their right to withdraw at any time without consequence. Consent was given with the explicit understanding that all data would be anonymized.

For interviews with hub representatives, participation has been agreed in the projects Consortium agreement. Nevertheless, verbal consent was also obtained and audio-recorded at the beginning of each session. This approach was chosen to reduce administrative burden and to suit the conversational, interactive nature of the interviews. For the Mobilisation and Mutual Learning (MML) workshops, participants provided consent via an online registration form, and an additional consent sheet was available at sign-in to confirm participation on the day of the event. For hub exchange activities and the quantification of project phases, consent was covered under the project’s Consortium Agreement.

All participants were adults and provided their own consent. They were informed that they could withdraw their data at any stage by submitting a written request to the corresponding author. Quotations and findings are presented in a way that fully safeguards the identity of individuals and organizations. Ownership of co-created knowledge and data was managed in accordance with the Consortium Agreement, which defines rights and responsibilities for data use and dissemination.

## Data availability

This study is based on qualitative data collected within Work Package 3 of the RuralBioUp project, covering seven countries: Italy, Romania, France, Ireland, Latvia, and the Czech Republic. The dataset consists of anonymized semi-structured interviews and workshop outputs.

To protect participant confidentiality, all interview data were fully anonymized before analysis. Personal identifiers were removed or replaced with codes, and any context that might allow indirect identification was carefully reviewed. Metadata describing the dataset follows FAIR principles, ensuring that it can be discovered and reused under appropriate conditions.

Access to the anonymized dataset can be requested by contacting the research coordinator, Margit Hofer ( hofer@zsi.at), at the Centre for Social Innovation (ZSI). Requests must include a written plan explaining the intended use of the data. Each request will be reviewed by the ZSI research coordinator in consultation with the RuralBioUp consortium to ensure compliance with ethical and legal requirements.

Data ownership and conditions of reuse are governed by the RuralBioUp Consortium Agreement. Approved users will be required to comply with the project’s data management and confidentiality provisions.

Further supporting materials, including interview guidelines and qualitative coding frameworks, are deposited in Zenodo:

Centre for Social Innovation. (2025). *RuralBioUp qualitative data sets.* Zenodo. https://doi.org/10.5281/zenodo.17579049 ^61^

All data are available under the terms of the Creative Commons Attribution 4.0 International License (CC-BY 4.0).

### Underlying data

This section presents detail all novel data collected and used as part of our article.

Details are hosted in the ZENODO repository from Margit Hofer, Martina Lindorfer, and Samire Gurgurovci in 2025 under https://doi.org/10.5281/zenodo.17579049. ^66^

This project contains the following underlying data:

• Data file 1. (semi-structured interview questionnaire guideline)
• Data file 2. (7 anonymised interviews)
• Data file 3 (Graph materials for qualitative analysis)
• Data file 4 (Graph on recommendations)

Data are available under the terms of Creative Commons Attribution 4.0 International.

### Extended data

Interview transcripts from the RuralBioUp projects were included in the data analysis.

## AI and generative AI use declaration

During the preparation of this manuscript, the authors used ChatGPT (version 4.0) for language editing and proofreading purposes. The AI tool was employed to review and refine the English language expression, grammar, and clarity of the written text while preserving the original meaning and scientific content. All substantive research content, analysis, interpretation of results, and conclusions presented in this work are entirely the authors’ own. The use of this generative AI tool was limited to language enhancement and did not contribute to the conceptual framework, data collection, analysis, or interpretation of findings.
