# Supplementary material for: Unlocking regional bioeconomy transitions: Co-creation, stakeholder engagement, and systemic change in rural Europe
Source: Open Res Eur. 2026 Jun 23;5:392. Originally published 2025 Dec 17. [Version 2] doi: 10.12688/openreseurope.21341.2 (PMC13550885; doi:10.12688/openreseurope.21341.2)
Supplement: Peer review history

# Reviewer report: Reviewer response for version 2

**DOI:** 10.21956/openreseurope.25756.r76543

**Published:** 2026-07-31

**Authors:** Hamid Balali

**Affiliations:** 1 New Mexico State University, Las Cruces, New Mexico, USA

This manuscript addresses an important, timely, and highly relevant topic: how regional bioeconomy initiatives can translate broad European bioeconomy strategies into locally relevant actions in rural areas. The study is particularly valuable because the transition toward a sustainable bioeconomy is inherently place-based and involves diverse actors, sectors, institutions, and knowledge systems. By examining stakeholder engagement, co-creation, innovation adoption, and the role of regional bioeconomy hubs, the manuscript addresses important practical and conceptual questions regarding how high-level bioeconomy strategies can be translated into effective local actions and how rural communities can participate in and contribute to this transition. The empirical focus on nine regional bioeconomy hubs established through the RuralBioUp project also provides an interesting opportunity to generate comparative insights across diverse rural contexts. The manuscript identifies several potentially important findings, including the importance of trusted local facilitators, established networks, locally grounded engagement, peer-to-peer learning, and the adaptation of innovations to existing practices and community values.

 I believe the study has the potential to make a meaningful contribution to the literature on rural bioeconomy transitions, place-based innovation, stakeholder engagement, and co-creation. However, several aspects of the conceptual framework, theoretical foundation, methodological transparency, and interpretation of the empirical findings should be strengthened to fully realize this potential and ensure that the conclusions are well supported by the evidence presented.

**1- The conceptual framework linking policy objectives to local implementation and systemic change could be further clarified.**

 The manuscript would benefit considerably from a clearer conceptual framework that explains the relationships among European-level strategies and policy objectives, the RuralBioUp project, regional hubs, local actors and institutional contexts, co-creation processes, actions and interventions, and potential systemic change. At present, these relationships remain somewhat implicit. As a result, it is difficult to determine exactly what the study is testing or explaining, what constitutes the unit of analysis, and how the empirical evidence supports the conclusions. The authors should explicitly clarify the relationship among European bioeconomy and rural development strategies, the European Green Deal, Horizon Europe, the RuralBioUp project, the establishment of Regional Hubs, local stakeholder engagement, co-creation processes, and the intended outcomes of innovation adoption and systemic change. The European Union increasingly positions the bioeconomy as an important component of the transition toward a circular, resource-efficient, and climate-neutral economy, while also emphasizing the role of rural areas, primary producers, businesses, and regional actors in this transition. The European Green Deal similarly emphasizes a transition toward a resource-efficient and competitive economy while ensuring that no person or place is left behind. In this context, the RuralBioUp project is explicitly designed to strengthen cooperation among regional actors and knowledge holders and to establish nine Regional Hubs across six EU countries to co-design and implement regional action plans and bio-based value chains.

 Therefore, the manuscript should make explicit **how the Regional Hubs function as a mechanism for translating high-level European policy objectives into place-based rural initiatives** **.** In particular, the authors should clarify what specific European-level strategies and policy objectives constitute the "high-level strategies" referred to in the Introduction, what types of local initiatives and innovations are expected to emerge from these strategies, and where the Regional Hubs are positioned within this broader policy-to-practice pathway. This relationship is particularly important because the RuralBioUp project itself conceptualizes the hubs as collaborative structures intended to involve regional stakeholders in developing contextually relevant bioeconomy value chains and innovation actions. The manuscript should therefore explain whether the hubs are primarily mechanisms for stakeholder coordination, co-creation and knowledge exchange, innovation adoption, institutional change, or all of these functions, and how these functions are expected to contribute to broader European objectives such as rural economic development, resource efficiency, climate neutrality, and the Green Deal. Making this policy and conceptual pathway explicit would substantially strengthen the manuscript's theoretical foundation and help readers understand the broader significance of the empirical findings beyond the individual project cases.

**2-** **The RuralBioUp project and its role in the study could be more clearly introduced.**

 The manuscript relies heavily on the RuralBioUp project as its empirical context, but the project itself is not sufficiently introduced or explained. According to the European Commission's CORDIS project information, RuralBioUp was a Horizon Europe project implemented from October 2022 to September 2025. The project aimed to strengthen cooperation among regional key actors and knowledge holders and to establish an inclusive and long-lasting ecosystem of Regional Hubs to support the mainstreaming of bio-based business models in rural areas. According to the project's original objectives, nine Regional Hubs were to be established across six EU countries, with the hubs co-designing and implementing nine Action Plans covering 18 bio-based value chains. The project also intended to support the Regional Hubs through mentoring, coaching, and training activities during the implementation of their Action Plans.

 These details should be explicitly integrated into the manuscript to provide readers with a clear understanding of the empirical and institutional context in which the study was conducted, rather than assuming that readers are familiar with the RuralBioUp project. In particular, the authors should clearly explain the objectives, structure, functions, and geographic scope of the Regional Hubs and clarify how they were expected to facilitate stakeholder collaboration, co-creation, innovation adoption, and the development of regional bioeconomy initiatives. This clarification is particularly important because the project's final reporting indicates that the nine Regional Hubs ultimately operated across seven countries-Italy, France, Ireland, Portugal, Latvia, Slovenia, and Romania-rather than the six countries specified in the original project objectives. The authors should therefore clarify the evolution of the project's geographic scope and provide a clear description of the nine hubs examined in this study.

**3-** **The theoretical framework could be further strengthened and clarified.**

 One important recommendation that could further strengthen the manuscript is to develop a more comprehensive and clearly articulated theoretical framework. The manuscript draws on several theoretically important concepts, including co-creation, place-based innovation, and systemic change. However, these concepts could be more clearly integrated into a coherent and well-articulated theoretical framework. For example, the manuscript states that co-creation is important but does not clearly explain what theory of co-creation guides the analysis. The authors should distinguish among related concepts such as stakeholder engagement, participation, co-production, co-design, co-creation, and collaborative governance. These concepts are not necessarily interchangeable. The manuscript should define precisely what "co-creation" means in this study and explain what distinguishes co-creation from conventional stakeholder consultation or participation.

**4-** **The description of the data sources and their coverage could be further clarified.**

 The manuscript states that data were collected from nine regional hubs; however, the data sources appear to include only seven semi-structured interviews. Providing a clear and transparent description of the methodology, ideally supported by an illustrative figure showing the relationship among the nine hubs, data sources, participants, and analytical procedures, would further strengthen the methodological rigor and transparency of the study. Clarifying questions such as whether all nine hubs were represented in the interviews, whether some hubs were represented only through documentary data, how many interviews were conducted for each hub, who the interviewees were (e.g., hub facilitators, project coordinators, or other stakeholders), and whether different stakeholder groups, such as farmers, SMEs, policymakers, researchers, and civil society organizations, were represented would help readers better understand the scope and diversity of the empirical evidence. It would also be helpful to clarify how many participants attended the mutual learning workshops and whether stakeholders from all nine hubs participated in both workshops. Providing these details with a figure would substantially strengthen the methodological clarity and help readers assess the scope, diversity, and representativeness of the data underlying the findings.

**5-** **The role of regional diversity and local context in the empirical analysis could be further explored.**

 The manuscript repeatedly emphasizes the importance of local context and place-based approaches. However, it is unclear whether the diversity among the nine regions was actually incorporated into the empirical analysis. The introduction refers to the challenge of translating "high-level strategies" into locally relevant initiatives. However, the manuscript does not clearly define what these high-level strategies are. The authors should identify and discuss the specific policy and strategic frameworks that motivate the project. For example, the manuscript could clarify how the bioeconomy transition relates to: European bioeconomy strategies; the European Green Deal; rural development policy; climate neutrality objectives; circular economy objectives; regional smart specialization strategies; relevant Horizon Europe missions or policy priorities. At present, the manuscript moves between these levels without clearly explaining the mechanisms linking them.

**References:**

 Reference: European Commission. (2026). *RuralBioUp: Empowering EU Rural Regions to scale-Up and adopt small-scale Bio-based solutions: The transition towards a sustainable, regenerative, inclusive and just circular bioeconomy*. CORDIS, European Commission. Grant Agreement No. 101060618. CORDIS RuralBioUp Project Information.

 European Commission. (2018). *A sustainable bioeconomy for Europe: Strengthening the connection between economy, society and the environment: Updated bioeconomy strategy*. COM (2018) 673 final. Brussels: European Commission. https://data.europa.eu/doi/10.2777/792130

 European Commission. (2019). *The European Green Deal*. COM(2019) 640 final. Brussels: European Commission.

 European Commission. (n.d.). *RuralBioUp: Empowering EU Rural Regions to scale-Up and adopt small-scale Bio-based solutions: The transition towards a sustainable, regenerative, inclusive and just circular bioeconomy*. CORDIS, European Commission.

If applicable, is the statistical analysis and its interpretation appropriate?

Not applicable

Is the study design appropriate and is the work technically sound?

Yes

Is the work clearly and accurately presented and does it engage with the current literature?

Yes

Are the conclusions drawn adequately supported by the results?

Partly

Are sufficient details of methods and analysis provided to allow replication by others?

Partly

Are all the source data and materials underlying the results available?

Partly

Reviewer Expertise:

Natural Resource Economics, Environmental Economics, Emission economics, Participatory System Dynamics, Local Knowledge and Stakeholder engagement, Climate change and Mitigation, Sustainability and system resilience, Multidisciplinary analysis of climate change adaptations

I confirm that I have read this submission and believe that I have an appropriate level of expertise to confirm that it is of an acceptable scientific standard.

# Reviewer report: Reviewer response for version 2

**DOI:** 10.21956/openreseurope.25756.r76343

**Published:** 2026-07-06

**Authors:** Francis Xavier Johnson

**Affiliations:** 1 Stockholm Environment Institute, Stockholm, Sweden

The authors seem to have addressed the review feedback, and I have no further comments to make.

If applicable, is the statistical analysis and its interpretation appropriate?

Not applicable

Is the study design appropriate and is the work technically sound?

Yes

Is the work clearly and accurately presented and does it engage with the current literature?

Yes

Are the conclusions drawn adequately supported by the results?

Yes

Are sufficient details of methods and analysis provided to allow replication by others?

Partly

Are all the source data and materials underlying the results available?

Partly

Reviewer Expertise:

conceptual formulation of bioeconomy visions and principles; interdiscipinary analysis of bio-based technologies; land-based mitigation; stakeholder engagement for climate resilient development scenarios

I confirm that I have read this submission and believe that I have an appropriate level of expertise to confirm that it is of an acceptable scientific standard.

# Reviewer report: Reviewer response for version 2

**DOI:** 10.21956/openreseurope.25756.r76344

**Published:** 2026-06-25

**Authors:** Carlos Zúñiga-Gonzáles

**Affiliations:** 1 National Autonomous University of Nicaragua, Leon, Nicaragua

I thank the authors for considering my observations in this revised version. Although the manuscript's main focus remains strictly limited to the internal governance of EU hubs, the inclusion of the final paragraph in the discussion enriches the work by placing European bioeconomic policies within the context of international trade, Article 4 of the SPS Agreement, and the challenges faced by producers in developing countries. I believe that the limitations and avenues for future research are now clearer and more comprehensive.

If applicable, is the statistical analysis and its interpretation appropriate?

No

Is the study design appropriate and is the work technically sound?

Yes

Is the work clearly and accurately presented and does it engage with the current literature?

Yes

Are the conclusions drawn adequately supported by the results?

Yes

Are sufficient details of methods and analysis provided to allow replication by others?

Yes

Are all the source data and materials underlying the results available?

Yes

Reviewer Expertise:

Bioeconomy, Productivity and Efficiency Analysis

I confirm that I have read this submission and believe that I have an appropriate level of expertise to confirm that it is of an acceptable scientific standard.

## References

[1] : Formulation of an innovative model for the bioeconomy. *PLOS ONE* .2024;19(11) : 10.1371/journal.pone.0309358 10.1371/journal.pone.0309358 PMC1153426239495794

[2] : TFP Bioeconomy Impact post Covid-19 on the agricultural economy. *PLOS ONE* .2023;18(11) : 10.1371/journal.pone.0288885 10.1371/journal.pone.0288885 PMC1065602637976256

[3] Sustainability in Latin America.2026; 10.1007/978-3-032-09787-3 10.1007/978-3-032-09787-3

# Reviewer report: Reviewer response for version 1

**DOI:** 10.21956/openreseurope.23081.r68714

**Published:** 2026-02-10

**Authors:** Carlos Zúñiga-Gonzáles

**Affiliations:** 1 National Autonomous University of Nicaragua, Leon, Nicaragua

It is recommended to deepen the discussion on the practical impact of reciprocity clauses on developing countries, as the text focuses heavily on the European and WTO perspectives but offers limited insight into how these standards act as technical barriers to trade for non-EU producers. The author should include a more detailed analysis of the mechanisms of "equivalence" versus "identity" of standards, clarifying whether third countries must strictly copy EU laws or if achieving similar welfare outcomes is sufficient. The manuscript would benefit from a clearer distinction between production standards that are visible in the final product and those that are not (non-product related PPMs), as this remains a core point of contention in WTO law. It is necessary to update the final references to ensure the inclusion of the most recent 2024 developments regarding European Commission legislative proposals that were recently postponed. Finally, the author should improve the transitions between the historical evolution section and the legal analysis of WTO cases to ensure the manuscript flows as a cohesive argument rather than isolated sections.

If applicable, is the statistical analysis and its interpretation appropriate?

No

Is the study design appropriate and is the work technically sound?

Yes

Is the work clearly and accurately presented and does it engage with the current literature?

Yes

Are the conclusions drawn adequately supported by the results?

Yes

Are sufficient details of methods and analysis provided to allow replication by others?

Yes

Are all the source data and materials underlying the results available?

Yes

Reviewer Expertise:

Bioeconomy, Productivity and Efficiency Analysis

I confirm that I have read this submission and believe that I have an appropriate level of expertise to confirm that it is of an acceptable scientific standard, however I have significant reservations, as outlined above.

## References

[1] Sustainability in Latin America.2026; 10.1007/978-3-032-09787-3 10.1007/978-3-032-09787-3

[2] : Formulation of an innovative model for the bioeconomy. *PLOS ONE* .2024;19(11) : 10.1371/journal.pone.0309358 10.1371/journal.pone.0309358 PMC1153426239495794

# Reviewer report: Reviewer response for version 1

**DOI:** 10.21956/openreseurope.23081.r66505

**Published:** 2026-01-28

**Authors:** Francis Xavier Johnson

**Affiliations:** 1 Stockholm Environment Institute, Stockholm, Sweden

The paper uses a qualitative approach that considers co-creation and stakeholder engagement with respect to three main questions: (1) the effectiveness of establishing regional bioeconomy hubs; (2) systemic elements of the co-creation process for such hubs; (3) inducing bio-innovation in traditional/conservative rural environments. The study draws on a cross-section of nine hubs across 7 EU countries.

 The research questions are sound and complement each other in understanding the dynamics around bio-innovation hubs. The study provides useful insights drawn from an analysis of these hubs through interviews, learning exercises, and workshops. There are nevertheless some shortcomings that should be addressed in order to improve the paper and enhance its value for the wider community of bioeconomy researchers and analysts.

 References: there are some parts in the Conceptual Framework section of the paper where a series of statements are provided, which, while appearing to logical, are unnacompanied by citations. For example, the last paragraph on LHS of page 6 has interesting and useful points but these are not backed up by the literature. The authors should check this and other sections, espceially in the Conceptual Framework section to be sure that they have grounded their approach well in the literature.

 Normative vs. Positive statements: the analysis and discussion mixes together normative and positive statements. This mixing is somewhat unavoidable due to the interactions between stakeholder-held beliefs and the processes used to uncover these beliefs, which are action or belief-oriented by their nature. Nevertheless, it would be helpful to be clear in the paper the arguments as to why some characteristics are inherently necessary or desirable - e.g. inclusiveness and/or diversity of stakeholders -- or why the authors assume that disruptive innovation is NEVER preferred. Clearly there will be some cases where society must adapt and change and some will be more willing to do so than others. A coalition of the willing is a part of achieving the economics of bio-based innovation just as with other types of innovation. The authors could screen the paper and take some care to avoid purely normative statements unless there is ample back-up for their validity.

 Economic Sustainability: related to the above, the activities of the hubs also need to be economically feasible and sustainable. Therefore, considerable effort for inclusion of groups who may ultimately not be willing or able to sustain the business activities may not always make sense. While economic sustainability alone is not sufficient, it is nevertheless one of the three legs of sustainability. Society can choose to protect vulnerable groups and this is indeed ethically and morally sound, but society cannot force enterprises to exist when the economics are too weak. Alternatively, we can design governance mechanisms to give greater voices to these groups and in some cases (but not all) the resulting enterprises will thrive. Cooperatives and local ownership (as noted by the authors) -- and other institutions that create a more balanced relation between how benefits, costs, and risks are shared or distributed -- offer a means to reconcile economic and social sustainability.

 Robustness: The importance of embedded peer networks and strong social capital is noted in the context of peers adopting practices observed to be successul (especially in paragraph 2 on page 9). At the same time, the authors note tendency towards risk aversion in rural environments. There is a saying that "if a technology works, you tell your neighbor, but if it fails you tell 10 neighbors." What this suggests is that there must also be mechanisms to insure robustness, i.e. even if the innovation or technology fails, there are ways to deal with it. It gives the adopters the confidence to try and fail. This may have been implieed but is not so explicit in the paper.

 Local facilitation and flexible governance: these are mentioned in the same bullet point in page 17 under Recommendations. Yet these are very different - adaptive or flexible governance is a different characteristic than local facilitation - i.e. the latter does not imply the former. These should be two separate recommendations (as they seemed to be on page 13).

 Supporting Methods and Data (I have labeled both as being made "Partly" available in questions above): although the authors provide supporting info on methods and data in terms of the interviews and analysis, there is no info on the actual activities of these hubs, and thus the various principles and conclusions espoused in the paper -- even though they seem reasonable and logical -- are rather abstract. It would be helpful to get more concrete info on the hubs themselves. In this way, the general principles, e.g. "learning by seeing," or "the need to see a real impact" could be understood in their tangible bioeconomy context. What did they see change, what impacts expected, how does it relate to their farming operations specifically, etc. A table with the various hubs that shows specific examples (even mentioning crops, resources, harvesting or conversion innovations, etc.) tied to some of these general principles - would help the reader to understand what specific issues that farmers and other actors are dealing with. There could be a supplementary table detailing key characteristics and experiences with the general and the specific laid out side by side where feasible and appropriate. Some of the info for the table might need to be taken from the actual activities at the hubs rather than from the interviews. This table does not have to be exhaustive and there is by definition no need for a one-to-one mapping since that was not part of the analysis, but it would be helpful with some simple characterisations of the actual activities at the hubs to make analysis and results less abstract.

If applicable, is the statistical analysis and its interpretation appropriate?

Not applicable

Is the study design appropriate and is the work technically sound?

Yes

Is the work clearly and accurately presented and does it engage with the current literature?

Yes

Are the conclusions drawn adequately supported by the results?

Yes

Are sufficient details of methods and analysis provided to allow replication by others?

Partly

Are all the source data and materials underlying the results available?

Partly

Reviewer Expertise:

conceptual formulation of bioeconomy visions and principles; interdiscipinary analysis of bio-based technologies; land-based mitigation; stakeholder engagement for climate resilient development scenarios

I confirm that I have read this submission and believe that I have an appropriate level of expertise to confirm that it is of an acceptable scientific standard, however I have significant reservations, as outlined above.

# Response

**Published:** 2026-05-07

**Authors:** Margit Hofer

**Affiliations:** Technology and Knowledge, Zentrum fur Soziale Innovation, Vienna, Vienna, Austria

Thank you for your valuable feedback. We have revised the manuscript to align with your suggestions. Below is a summary of the key changes: Clearer Distinctions Between

 Normative and Positive Statements:

 We have clarified the distinction between normative and positive statements throughout the paper, particularly in the Conclusions and Recommendations. For example, we revised "Inclusiveness is crucial for bioeconomy hubs" to indicate that while inclusiveness was associated with success, it may not always be essential in every context. We also noted that disruptive innovation is one potential approach but incremental or co-adaptive innovation was more commonly successful in the hubs observed.

 Economic Sustainability and Robustness:

 We expanded the discussion on economic sustainability and robustness to reflect the study’s findings. We now highlight market-driven models, cooperative ownership, and public-private partnerships as key financial strategies for long-term viability. We also emphasize the importance of robustness, with hubs incorporating safe experimentation and peer learning showing greater resilience.

 Clarity in the Recommendations:

 We separated economic sustainability and robustness into distinct recommendations, providing actionable strategies for designing financial models that balance economic, social, and environmental goals, and fostering resilience through adaptive governance. We believe these revisions effectively address your feedback, ensuring the paper reflects the study’s findings while offering actionable recommendations for future bioeconomy initiatives.

 Thank you again for your constructive feedback!
